# 3D Microstructure Effects in Ni-YSZ Anodes: Prediction of Effective Transport Properties and Optimization of Redox Stability

**DOI:** 10.3390/ma8095265

**Published:** 2015-08-26

**Authors:** Omar M. Pecho, Ole Stenzel, Boris Iwanschitz, Philippe Gasser, Matthias Neumann, Volker Schmidt, Michel Prestat, Thomas Hocker, Robert J. Flatt, Lorenz Holzer

**Affiliations:** 1 Institute of Computational Physics, Zurich University of Applied Sciences, Winterthur 8400, Switzerland; E-Mails: o.stenzel@gmx.de (O.S.); michel.prestat@gmail.com (M.P.); hoto@zhaw.ch (T.H.); holz@zhaw.ch (L.H.); 2 Institute for Building Materials, ETH Zurich, Zurich 8093, Switzerland; E-Mail: flattr@ethz.ch; 3 Hexis SA, Winterthur 8404, Switzerland; E-Mail: Boris.Iwanschitz@buderus-steel.com; 4 Scientific Center for Optical and Electron Microcopy (ScopeM), ETH Zurich, Zurich 8093, Switzerland; E-Mail: philippe.gasser@scopem.ethz.ch; 5 Institute of Stochastics, Ulm University, Ulm 89069, Germany; E-Mails: matthias.neumann@uni-ulm.de (M.N.); volker.schmidt@uni-ulm.de (V.S.)

**Keywords:** cermet, degradation, microstructure, tomography, conductivity, solid oxide fuel cells, Ni-YSZ, redox cycling

## Abstract

This study investigates the influence of microstructure on the effective ionic and electrical conductivities of Ni-YSZ (yttria-stabilized zirconia) anodes. Fine, medium, and coarse microstructures are exposed to redox cycling at 950 °C. FIB (focused ion beam)-tomography and image analysis are used to quantify the effective (connected) volume fraction (Φ_eff_), constriction factor (β), and tortuosity (τ). The effective conductivity (σ_eff_) is described as the product of intrinsic conductivity (σ_0_) and the so-called microstructure-factor (*M*): σ_eff_ = σ_0_ × *M*. Two different methods are used to evaluate the M-factor: (1) by prediction using a recently established relationship, *M*_pred_ = εβ^0.36^/τ^5.17^, and (2) by numerical simulation that provides conductivity, from which the simulated M-factor can be deduced (*M*_sim_). Both methods give complementary and consistent information about the effective transport properties and the redox degradation mechanism. The initial microstructure has a strong influence on effective conductivities and their degradation. Finer anodes have higher initial conductivities but undergo more intensive Ni coarsening. Coarser anodes have a more stable Ni phase but exhibit lower YSZ stability due to lower sintering activity. Consequently, in order to improve redox stability, it is proposed to use mixtures of fine and coarse powders in different proportions for functional anode and current collector layers.

## 1. Introduction

Solid oxide fuel cells (SOFC) are electrochemical conversion devices that produce electricity directly from oxidizing a fuel (e.g., natural gas, biogas, H_2_). The advantages of SOFCs are high efficiency, fuel flexibility, and low emissions (pollutants and noise) [1,2]. However, the materials are exposed to harsh operating conditions, which include high chemical potentials between anode (reducing atmosphere) and cathode (oxidizing) in combination with high temperatures (750–950 °C). These harsh conditions may reduce the fuel cell lifetime. Especially in conventional Ni-based anodes, so-called redox cycles that occur during shutdown and restart procedures often lead to a harmful degradation. In the present study we thus investigate the relationship between topological parameters (e.g., tortuosity, constrictivity) with effective anode properties (e.g., ionic or electrical conductivities). 

The electrochemical performance of Ni-YSZ anodes depends on the interplay between different transport processes and the electrochemical reactions at the triple phase boundary (TPB). Oxygen ions are transported through the ion-conducting yttria-stabilized zirconia (YSZ), electrons through the electron-conducting Ni, and gas species through the pores, while the electrochemical reaction primarily occurs near the TPB. The fuel oxidation at the TPB includes numerous intermediate steps [3], which can be summarized into the following net reaction:

(1) $$H_{2}+O^{2-}\to H_{2}O+2e^{-}$$

The associated charge transport and charge transfer reactions provide specific contributions to the area specific resistance (ASR). The ASR contribution of each process depends on both the relevant intrinsic property (e.g., intrinsic ionic conductivity of YSZ) and on the corresponding morphological characteristics (e.g., volume fraction and tortuosity of YSZ). The combination of intrinsic properties and microstructural characteristics results in a homogeneous effective macro-property (e.g., effective ionic conductivity).

One way to improve the effective properties and the associated electrode performance is to tailor the microstructure. However, the optimization of electrode microstructures is challenging because of the complex interplay between the various processes taking place [4]. The different properties cannot be manipulated independently from each other. In literature, strong focus was laid specifically on the improvement of the electrochemical activity, which correlates with the TPB length (*i.e.*, the number of electrochemically active sites). Therefore, the tendency is to develop finer-grained microstructures with higher TPB length [5]. Nevertheless, in order to ensure that the reaction from Equation (1) occurs throughout the anode, all three phases joining at the TBP must constitute a connected network. Thus, for a high anode performance the microstructure must also be optimized in terms of connectivity and the effective transport properties of all three phases (Ni, YSZ, pores). In addition, it is not only the initial microstructure that is important for the successful application of the fuel cell technology. For example, electrolyte-supported SOFCs with an open system configuration undergo several reduction-oxidation (redox) cycles, which often cause degradation of the microstructure and an associated loss of electrochemical performance. Hence, redox stability is an additional quality criterion for such SOFC anodes [6].

Redox cycles involve the reoxidation of Ni to NiO and then re-reduction back to Ni. The reaction is accompanied by a volume change of about 70% [5]. The oxidation kinetics are controlled mainly by the outward diffusion of Ni and inward diffusion of oxygen. During reduction, additional pores are formed and, thus, the overall kinetics are not diffusion-controlled, but they rather depend on the kinetics of surface reactions (e.g., formation of mobile hydroxide species). Repeated redox cycling causes an increase of anode thickness, which can be considered macroscopic swelling or irreversible expansion. It is generally attributed to the fact that volume expansion during reoxidation is not fully reversible during re-reduction, which leads to an increase in porosity throughout the entire anode microstructure [7]. Nevertheless, on the grain scale (*i.e.*, locally), the volume expansion of Ni particles may be reversible after re-reduction. Aside from the macroscopic swelling, a redistribution and coarsening of Ni also occurs during redox cycling [6]. The mobilization of Ni is strongly enhanced by the formation of Ni(OH)_2_ species, which have much higher gas saturation pressures compared to Ni and NiO [8]. Especially at high temperatures (>700 °C), the redistribution of Ni (via Ni(OH)_2_) can become very prominent. Thereby, small Ni grains tend to agglomerate in order to minimize surface energy. The coarsening leads to a degradation of electrical conductivity, and also to a loss of TPB and associated electrochemical activity.

In contrast to Ni, YSZ is generally considered the rigid ceramic backbone of the anode, which provides mechanical stability [5,6,9]. However, it was shown that the ionic conductivity decreases after redox cycling [6], which may be related to the crystallographic transformations in YSZ at elevated temperatures. Alternatively, it may be due to the fact that the elevated mobility of ionic species (e.g., Zr^4+^) under redox conditions may lead to changes of both the YSZ microstructure and its intrinsic conductivity [10,11,12]. Nonetheless, YSZ can also be affected by the stress induced either by the Ni coarsening or by the subsequent reoxidation, which creates mechanical stresses along the weak points of the ceramic backbone [6].

In order to study the influence of anode microstructure on the performance and redox stability, the relevant topological parameters must be quantified. For this purpose, three-dimensional (3D) image analysis is required to gain the necessary information on phase connectivity, tortuosity, constrictivity, and active TPB length. Using focused-ion beam scanning electron microscopy (FIB-SEM) [13,14,15,16,17,18,19,20] and X-ray tomography [21,22,23,24,25,26], it is possible to obtain 3D microstructure data which can be used to quantitatively describe the anode topological characteristics. Moreover, information gained from 3D microstructure analysis can be used to design anode microstructures with improved effective properties and redox stability.

In principle, the controlled materials design includes two steps: (1) establishing experimental methods by which the topological parameters can be influenced in the fabrication process (e.g., deposition method, composition, powder fineness, sintering conditions) [27,28,29,30,31,32,33]; and (2) formulating relationships between microstructure properties and effective material properties. In the past, the second relationship was not established on a quantitative level. However, recently, this gap was filled by an approach using virtual materials design (VMT) [34], thereby creating a large number of virtual 3D microstructures by means of spatial stochastic simulations. The corresponding effective properties (*i.e.*, conductivities) were then determined with numerical transport simulations. Using statistical analysis (*i.e.*, error minimization) an empirical equation describing the relationship between topological parameters and the effective transport property was obtained (see Equations (3) and (4)). The derivation of this empirical equation is discussed in the next section.

In general, the influence of microstructure on the effective transport property can be given with the following simple relation:

(2) $${\text{σ}}_{eff}=\;{\text{σ}}_{0}M$$

where the M-factor (*M*) stands for microstructure influence, and *σ*_eff_ and *σ*_0_ are the effective and intrinsic transport properties, respectively [35]. The M-factor includes the topological parameters, which are relevant for transport:

(3) $$M=\;\frac{{\left(\text{ϕ}P\right)}^{a}{\text{β}}^{b}}{{\text{τ}}^{c}}$$

Thereby, Φ is the volume fraction of the transporting phase. *P* is the percolation factor. Φ and *P* together combine into Φ_eff_, which is the effective phase volume that contributes to transport. The constriction factor (β) introduced by Petersen [36] can be interpreted as the ratio between the average sizes of the bottlenecks and the bulges (*r*_min_/*r*_max_)^2^ [37], and describes the bottleneck effect [38,39,40]. Tortuosity (τ) is a statistical expression for the windedness of transport pathways [41]. There are a few definitions of tortuosity. For example, the work with VMT [34] uses the so-called geometric tortuosity (with all transport pathways following a median axis skeleton), while another work [42] uses geodesic tortuosity (with the shortest transport pathways through the voxel-space of a given phase). The exponential parameters *a*, *b*, and *c* are determined empirically with the above-mentioned VMT approach and with statistical error minimization. Following the work of Stenzel *et al.* [42] (where *τ* represents geodesic tortuosity), the empirical relation in Equation (3) takes the form:

(4) $$M_{pred}=\;\frac{{\text{ϕ}}_{eff}{\text{β}}^{0.36}}{{\text{τ}}^{5.17}}$$

Whenever all the necessary topological parameters are known, the M-factor can be determined from Equation (4) and the effective transport property from Equation (2). One advantage of using such empirical relationships is the possibility to predict material properties that are difficult to access experimentally. For example, in SOFC anodes, this approach opens new possibilities to gain information on the effective ionic conductivity in YSZ, which is usually unknown or expensive to measure. More details on the methods to extract the required topological parameters from tomographic data can be found in previous works [37,43,44]. The topological parameters are also described in the experimental section below.

In the present study, we use the above-mentioned methods to quantify topology and to predict effective transport properties in order to investigate microstructure effects in Ni-YSZ anodes. A special focus is given to the degradation mechanism upon redox cycling and on the redox stability. Ni-YSZ anodes of varying microstructure coarseness (fine, medium, coarse) before and after redox cycling at 950 °C are investigated. The microstructures of fresh anodes and the corresponding electrical and ionic conductivities are compared to each other. Then the influence of redox cycling on anode properties is documented in order to unravel the degradation mechanisms. We use the established empirical relationship between microstructure parameters and effective conductivity (resulting in *M*_pred_) and apply the predictive model to different SOFC anode microstructures. Also, in this work, we determine the M-factor by numerical simulation of conduction (*M*_sim_), using the voxel-based tomography data as a structural input (with GeoDict software). The comparison of *M*_sim_ and *M*_pred_ can be considered as a validation of the empirical prediction approach.

Thus far, the redox degradation was mainly described by qualitative observations (e.g., Ni agglomeration) or by measuring parameters, which cannot be linked directly to an effective property (e.g., average particle size). With the proposed approach where topological parameters are linked with conductivity on a quantitative level, the diagnosis of degradation effects becomes more specific. For example, the loss of conductivity can be directly related to the change of bottleneck dimensions and associated constrictivity, or the change of the (average) length of the transport pathway and the associated geodesic tortuosity. As an outcome, the detailed analysis of microstructure parameters leads to a more differentiated interpretation of redox degradation. Finally, this knowledge leads to defining fabrication guidelines for the improved redox stability of Ni-YSZ anodes.

## 2. Experimental

The procedures for fabrication and electrical characterization of Ni-YSZ anodes are described in great detail by Iwanschitz *et al.* [45,46]. The following Section 2.1 and Section 2.2 represent a short summary of the experimental procedures, followed by a description of the applied imaging and image analysis techniques Section 2.3 and Section 2.4.

### 2.1. Anode Fabrication and Redox Cycling 

Three different anodes with fine, medium, and coarse microstructures are produced by screen printing, oxidized sintering, and subsequent reduction. To vary the microstructure, three different powders (fine, medium, coarse) of 8YSZ (Mel Chemicals, purity > 99%) are mixed always with the same powder of NiO (J.T. Baker, purity > 99%) to form Terpineol-based slurries. The coarse powder is produced by calcination of the fine powder (Mel Chemicals). The particle size distributions of the raw NiO and YSZ powders measured using a Horiba LA-920 laser scattering analyzer can be found in the supplementary information (Table S1). The ratio of the powders is chosen such that the solid volume fraction (Ni:YSZ) after reduction is 40:60. The Terpineol-based slurries are then screen-printed onto 3YSZ substrates (Nippon Shokubai, 140 μm) to give a 12 × 12 mm^2^ cell dimension. The layer thickness of the fine, medium, and coarse anodes are 20, 25, and 26 μm, respectively. The anodes are sintered in air at 1350 °C for four hours after screen-printing. Subsequent reduction is performed at 950 °C.

Degradation experiments are done by exposing the anodes to eight redox cycles in a tube furnace at 950 °C. For each cycle, reoxidation is carried out for 30 min and the re-reduction is carried out by ramping H_2_ flow for 5 min, with equilibration time of at least 30 min.

### 2.2. Electrical Conductivity Measurements 

Four-point conductivity measurements are performed on 8.8 × 21 mm^2^ samples cut from anode half-cells. The samples are placed in an alumina tube and contacted with Pt wire. The conductivity measurements are done at 950 °C with a gas flow of 200 mL/min using forming gas (5% H_2_/95% N_2_). The Nernst potential is kept constant at 1.1 V during the measurements and the gas is not humidified in order to exclude ill-controlled degradation effects from water during the redox cycling. Between each redox cycle, the alumina tube is flushed with N_2_ for one hour and the anodes are oxidized in airflow of 100 mL/min.

### 2.3. Image Acquisition by FIB-Tomography and SEM

For microstructure analysis, polished cross-sections are prepared from the samples that were used for four-point conductivity measurements. The porous anodes are first impregnated with a low-viscosity resin and then polished on textile substrates with 1, 3, and 6 μm diamond suspensions. Focused ion beam (FIB)-SEM tomography is performed with a Helios Nanolab 600i (FEI) with Ga liquid metal ion source. FIB tomography includes a repetitive procedure of alternating ion sectioning and SEM imaging. The serial sectioning is done with an ion beam current of 2.5 nA and an accelerating voltage of 20 kV. For serial SEM imaging, good contrast settings are achieved with the so-called through-the-lens detector (TLD) at 2.0 kV accelerating voltage and 0.69 nA beam current.

### 2.4. Quantitative 3D Microstructure Analysis

**Figure 1:**
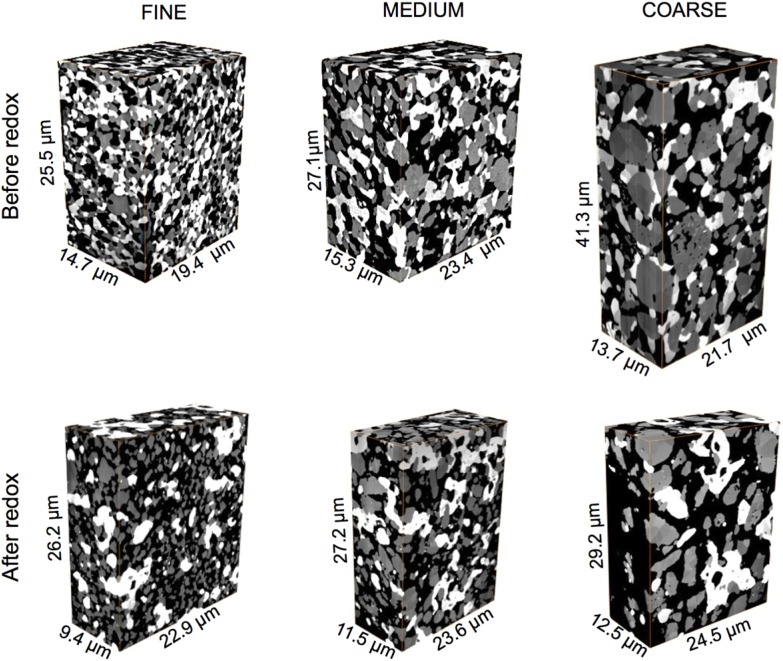
3D microstructures (FIB-tomography) of Ni-YSZ anodes with increasing coarseness from left to right. Top row is before and bottom row is after redox cycling at 950 °C. Pores (**black**), YSZ (**gray**), and Ni (**white**). Significant Ni agglomeration is visible in all samples after redox cycling.

FIB-tomography provides image stacks, which typically consist of 500 to 1000 two-dimensional (2D) images. Prior to the quantification of transport-relevant topological parameters the stacks have to be pre-processed. This procedure includes alignment (3D reconstruction), selecting a region of interest (cropping), noise filtering, and segmentation (*i.e.*, identifying Ni, YSZ, and pore phases). In our workflow we use Fiji [47], including some home-made plug-ins and Avizo (version 8.1.1) [48] software packages. Further details on image processing can be found in our previous study [37]. 3D reconstructions of the fine, medium, and coarse Ni-YSZ anodes before and after redox cycling are shown in Figure 1. The corresponding dimensions of the tomographic data are summarized in the supplementary materials (Table S2).

Particle and pore size distributions are determined by a method called continuous phase size distribution (c-PSD), which was developed by Münch and Holzer [44]. Contrary to conventional PSD methods, which are based on size statistics of discretized objects, c-PSD treats each phase as a continuum. A cumulative continuous size distribution is achieved by incrementally decreasing the size of spheres, which cover a particular phase volume and are completely contained in this volume. The radius corresponding to 50 vol % in the cumulative c-PSD curve (*r*_50,max_) is considered a statistical measure for the average size of the phase. Hence, this so-called *r*_50,max_ represents the average size of bulges in a percolating network of either a solid or pore phase.

Based on 3D information from tomography mercury intrusion porosimetry (MIP), a method that describes pore size distributions by pressure infiltration can be simulated. According to the so-called Washburn equation [49], the capillary radius is inversely proportional to the applied pressure. A cumulative MIP-PSD is thus obtained by linking the infiltrated volume with the corresponding pressure and capillary radius. It is currently well accepted that the MIP-PSD mainly captures the size of bottlenecks, which dominate the infiltration process [50]. Here we apply the method of Münch [35], which uses the same principle as the c-PSD analysis. However, it adds the constraint that the incrementally added volume must be connected with the plane from where the infiltration starts. A cumulative MIP-PSD curve is thereby obtained by incrementally decreasing the sphere radius, which corresponds to increasing pressures in the experimental MIP analysis. The “inkbottle effect” governs the measurement like in the experimental counterpart such that the dimensions of the bottlenecks limit the intrusion. Unlike the c-PSD, MIP-PSD measures the size of the bottlenecks (*r*_50,min_) once the breakthrough pressure is passed. Both methods can be applied to describe size distributions of pores as well as solid phases.

The constriction factor (β) is given by the ratio of the cross-sectional areas of the bottlenecks to the bulges (*A*_min_/*A*_max_) and further simplification leads to the following relation:

(5) $$\text{β}=\;\left(\frac{\text{ϐ}r_{min}^{2}}{\text{ϐ}r_{max}^{2}}\right)=\;{\left(\frac{r_{min}}{r_{max}}\right)}^{2}$$

This concept was adapted to describe bottleneck effects in disordered microstructures (e.g., fuel cell anodes) [37]. For this purpose, the results from c-PSD (*r*_50,max_) and MIP-PSD (*r*_50,min_) are used to describe the average sizes of bulges and bottlenecks, respectively. By Equation 5 we obtain the constriction factor (β), which can be plugged into Equation (4). Tubes of constant radius have a constriction factor of 1, while in disordered microstructures of varying radii the values are <1.

Geodesic tortuosity, τ_geod_ (simply τ) is defined as the ratio of geodesic distance (*d*_geod_) and material thickness (*l*). In general, the geodesic distance can be interpreted as the shortest path length from one side of the material to the other side through the transporting material phase. For statistical accuracy, we consider the mean value of geodesic distances (shortest path lengths) for numerous starting points. More precisely, any pixel in the so-called in-plane, which belongs to the transporting phase, is considered a starting point for shortest path analysis. The shortest path lengths are calculated using Dijkstra’s algorithm [51]. Therefore, the image is interpreted as a graph in which each voxel of the transporting phase is a node and all the nodes are connected whenever the corresponding voxels are connected with respect to the 26-neighborhood. Note that the geodesic tortuosity should not be mixed up with the geometric tortuosity. For the latter, Dijkstra’s algorithm is applied to a skeleton graph of the transporting phase. The two methods were compared in a recent study by Stenzel *et al.* [42] and it was concluded that the geometric definition overestimates the tortuosity, while the geodesic definition captures tortuosity with respect to the conductive transport processes more adequately.

### 2.5. Conductivities and M-Factor Simulation

Equations (2)–(4) describe a method to predict effective conductivities by using quantitative microstructure analysis and the M-factor (*M*_pred_), respectively (see Equation (4)). As an alternative approach, the effective ionic and electrical conductivities are also assessed by numerical simulation using the software GeoDict (version 2014 Rev. March 2015) [52]. The mathematics involved in the simulation are extensively described by Wiegmann and Zemitis [53]. The simulations of electrical and ionic conductivities were done along *x*, *y*, and *z* directions, using the real tomographic data as input. It should be noted that for the case where the phase of interest takes an intrinsic conductivity value of 1, (while all other phases are set to 0), the obtained conductivities also represent the M-factor (*i.e.*, *M*_sim_). The results from the simulation (*M*_sim_) are subsequently compared to the results from the predicted M-factor from the microstructure analysis (*M*_pred_). The dimensions of the tomographic 3D data used in the simulations are summarized in Table S3 in the supplementary materials.

## 3. Results

### 3.1. Qualitative Description of Microstructures

The initial microstructures are strongly influenced by the sintering process and the initial reduction conditions. It is important to note that the morphological characteristics of Ni and the pores are influenced by the coarseness of the YSZ powder. During the anode fabrication only the starting particle size of the YSZ powder is varied (fine, medium, coarse) while the particle size of NiO is the same for all the samples.

**Figure 2:**
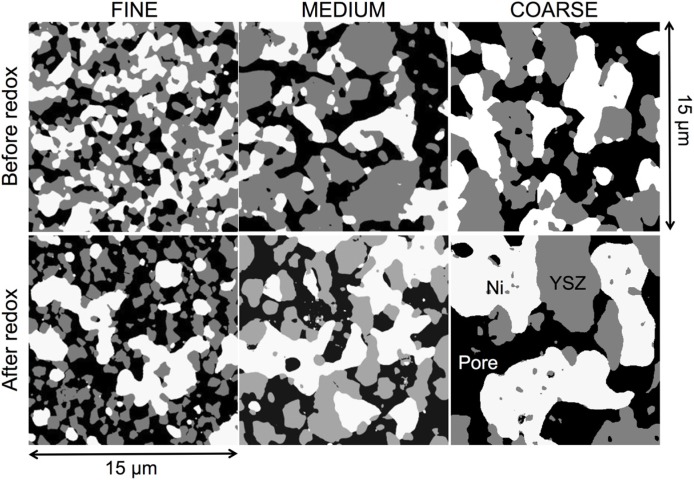
2D slices of segmented 3D images of all the Ni-YSZ samples investigated in this study. Three different YSZ powders (fine, medium, and coarse) are used for composite anode fabrication. In the initial microstructures (**top row**), Ni (**white**) and pores (**black**) follow the size of the YSZ (**gray**) starting particle size. Redox cycling (**bottom row**) leads to significant coarsening and agglomeration of Ni.

The visual inspection of 3D reconstructed images in Figure 1 and Figure 2D segmented images in Figure 2 show that upon sintering and reduction, the microstructures of Ni and pores are adapting to the microstructure of YSZ. This adaptation already starts during sintering in air (before reduction), where NiO changes its size according to the coarseness of the YSZ (not shown here). Hence, the anodes fabricated with coarser YSZ also exhibit coarser microstructures of Ni and pore phases. However, the inspection of 3D images also indicates that Ni and YSZ have different characteristics. YSZ appears to be more granular with distinct sinter necks, whereas Ni forms a more continuous network with a greater structural heterogeneity.

The microstructure degradation related to redox cycling can be observed in Figure 2 by comparing the top (before) and bottom rows (after redox). Significant Ni coarsening is observed after redox cycling. Ni tends to form agglomerated patches, which are particularly strong in the fine anode. In contrast, the microstructure of YSZ seems to withstand redox alterations. This is compatible with the interpretation of YSZ being the rigid backbone of Ni-based anodes. An additional aspect of redox degradation is the increase of intergranular porosity, which is again stronger in the fine anode. On a macroscopic scale, the increase of the pore volume fraction may result in an overall swelling. Consequently, the swelling will invoke a relative decrease of the solid volume fractions.

### 3.2. Electrical Conductivity Measurements

**Figure 3:**
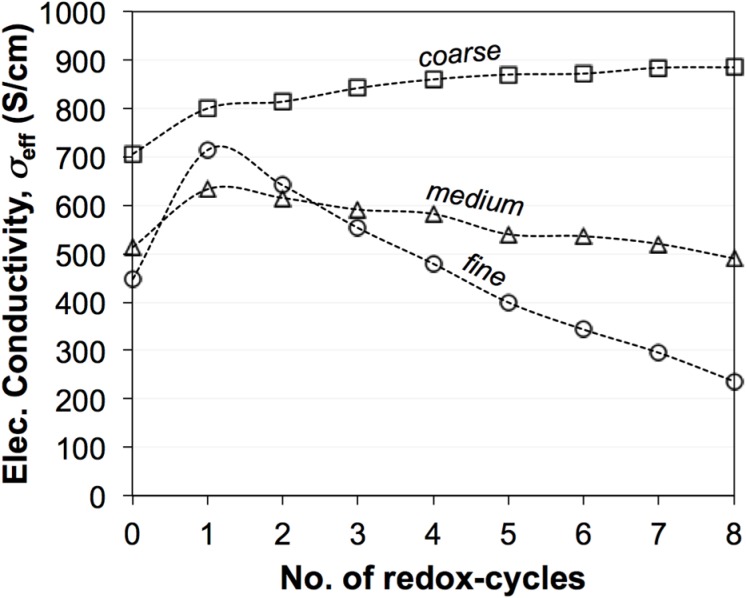
Evolution of electrical conductivities (σ_eff_) of Ni-YSZ during exposure to eight redox cycles at 950 °C based on the work of Iwanschitz [45]. Linear trends of degradation are observed between the first and eighth cycles. σ_eff_ of the fine anode is strongly degrading. In the medium-grained anode, σ_eff_ is slightly decreasing. In the coarse anode, σ_eff_ is improving. The differences in the initial values and in the curing behavior during the first cycle are not well understood.

Figure 3 shows the evolution of electrical conductivities (σ_eff_) during eight redox cycles at 950 °C [45]. The fine sample exhibits the greatest degradation of σ_eff_, while the coarse sample even shows an improvement in σ_eff_. Significant differences are measured for the initial values (zeroth) and for the first redox cycle. These initial differences are not well understood. They may be related to experimental issues such as contacting and/or to microstructure reorganization after reduction. In contrast, the linear trends from the first to the eighth cycle are interpreted as typical redox behavior. Therefore, in the discussion, the values of σ_eff_ after the first redox cycle are taken as the reference electrical conductivity (σ_eff_,_before_). The last redox cycle (eighth) is taken as the electrical conductivity after redox cycling (σ_eff_,_after_). The data set in Figure 3 will be compared to predicted transport properties, which are based on topological parameters obtained from 3D image analysis (see Equations (3) and (4)).

### 3.3. Effective Volume Fractions (Φ_eff_, ε_eff_) and Percolation (P)

**Figure 4:**
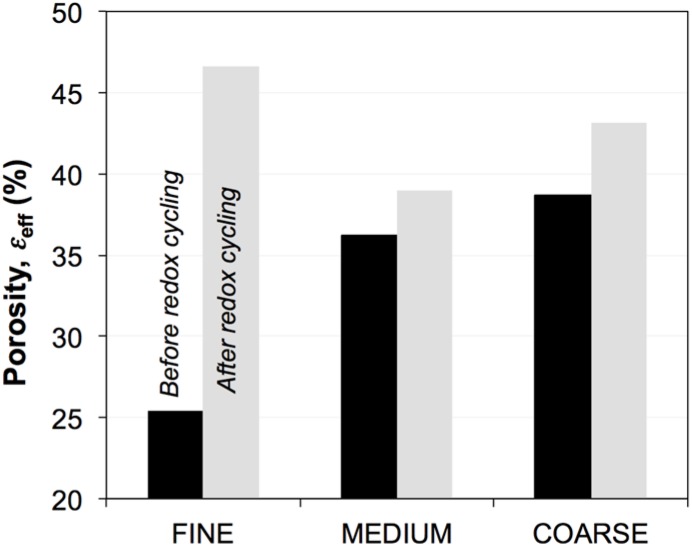
Porosity (ε) in Ni-YSZ anodes. In fresh samples, porosity increases from fine to coarse. After sintering, the fine anode is relatively dense. Redox cycling then leads to a significant increase of porosity in the fine anode.

**Figure 5:**
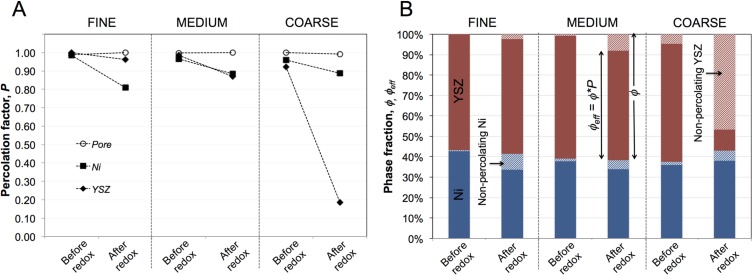
(**A**) Percolation factors (*P*) of the different phases and (**B**) solid volume fractions (Φ, Φ_eff_) in the six Ni-YSZ anodes. *P* describes the interconnected portion of a phase. (**A**) After redox cycling the percolation factors of YSZ decrease significantly in the coarse anode. The percolation factors of Ni decrease only moderately (–10% to –20%). (**B**) The ratio Ni:YSZ is similar in all six samples (40:60 vol %); however, the effective volume fractions (interconnected portions) of YSZ and Ni show partially strong variations upon redox cycling due to changes of *P* (e.g., YSZ coarse and Ni fine).

In the subsequent Section 3.3, Section 3.4 and Section 3.5, we present results from the quantitative 3D analysis with a strong focus on parameters that are relevant for the effective transport properties. The porosities (ε_eff_) of the six investigated SOFC anodes are shown in Figure 4, and the ratios of the solid volume fractions (Φ_Ni_/Φ_YSZ_) are presented in Figure 5. Before exposure to redox cycling, the porosity increases from fine (25 vol %) to medium (36 vol %) to coarse (39 vol %). During redox cycling, the porosity increases in all three microstructures and is most pronounced in the sample with the fine microstructure (83 vol % increase) compared to medium and coarse samples (8 and 12 vol %).

The ratios Φ_Ni_/Φ_YSZ_ are similar for all six samples before and after redox cycling (40:60 vol %) as shown in Figure 5B. However, considerable differences occur with respect to the effective volume fraction (Φ_eff_), which is defined as the product of the total volume fraction (Φ) multiplied by the percolation factor (*P*). The latter parameter describes the fraction of a phase, which forms a connected network, and it can be obtained from the MIP-PSD analysis (Figure 5A). Before exposure to redox-cycling, the percolation factors are close to 1. The cumulated percolation factors for Ni, YSZ, and pores together comprise 0.991, 0.985, and 0.962 in fine, medium, and coarse microstructures, respectively. After redox cycling, the percolation factors of YSZ and Ni decrease. This change is particularly strong for YSZ in the coarse sample, where the percolation factor drops to 0.18. For the Ni phase the changes are largest in the fine sample, where the *P* drops from 0.99 to 0.80 (compared to 0.90 in the coarse sample). In all samples the *P* of pores remains close to 1 before and after redox cycling.

### 3.4. Phase Size Distributions (PSD), Average Radii (r_50,max_, r_50,min_), and Constriction Factor (β)

**Figure 6:**
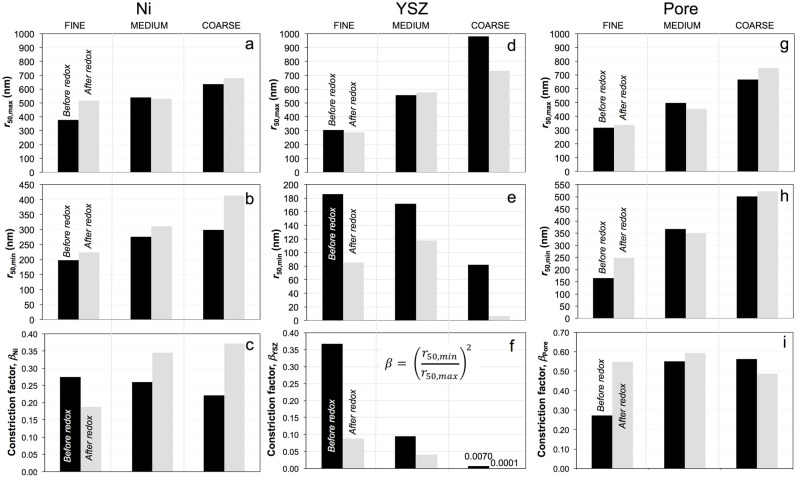
(**a**,**d**,**g**) Average sizes of bulges (*r*_50,max_ from c-PSD); (**b**,**e**,**h**) bottlenecks (*r*_50,min_ from MIP-PSD); and (**c**,**f**,**i**) constriction factors (β) for Ni, YSZ, and pores in composite anodes, before (black) and after redox cycling (gray). Ni: The β_Ni_ in the fine sample decreases due to the growth of bulges (*r*_50,max_ ↑) while the βNi in medium and coarse samples increase due to the widening of the bottlenecks (*r*_50,min_ ↑). YSZ: Upon redox cycling, the sizes of the bulges remain nearly constant while the bottlenecks are shrinking (*r*_50,min_ ↓). Consequently, all the βYSZ decrease after redox cycling. Pore: For medium and coarse samples all parameters remain stable during redox cycling. In the fine sample the bottlenecks expand (*r*_50,min_ ↑), which results in a higher β_Pore_ after redox cycling.

The c-PSD and MIP-PSD curves for Ni in fine and coarse anodes are shown in the supplementary materials (Figure S1). The PSDs for medium-grained anodes are not shown for simplicity. The average radii (*r*_50,max_, *r*_50,min_) and associated constriction factors of Ni, YSZ, and the pores are summarized in Figure 6. The *r*_50,max_ of Ni particles in fine, medium, and coarse anodes are 378, 540, and 636 nm (Figure 6a) and the average bottleneck sizes (*r*_50,min_) are 204, 275, and 300 nm (Figure 6b). Although the bulges and bottlenecks increase from fine to coarse, the constriction factor (β_Ni_) exhibits a slightly decreasing trend from 0.29 (fine) to 0.22 (coarse) (Figure 6c).

Upon redox cycling, the average size of Ni (*r*_50,max_) increases from 378 nm (before) to 517 nm (Figure 6a). For medium and coarse microstructures, the c-PSDs and associated average Ni sizes (*r*_50,max_) remain almost unchanged. In contrast, the Ni bottlenecks (*r*_50,min_) in all the samples exhibit an increase upon redox cycling with the most significant change for coarse anodes (Figure 6b). Consequently, in the fine sample the constriction factor (β_Ni_) decreases significantly from 0.29 to 0.18 (Figure 6c) mainly due to grain growth (increase of *r*_50,max_). In the medium and coarse samples β_Ni_ increases to 0.34 and 0.37 due to the opening of the bottlenecks (Figure S1B, increase of *r*_50,min_) after redox exposure.

As illustrated in Figure 6d, the YSZ phase exhibits the increasing size of the bulges from fine to coarse before redox cycling (*i.e.*, 360, 557, and 981 nm for the fine, medium, and coarse samples, respectively). This is accompanied by decreasing average radii of the bottlenecks (195, 172, and 82 nm, Figure 6e). Consequently, the constriction factor (β_YSZ_) before redox cycling decreases from 0.37 to 0.007 (fine to coarse). The ceramic phase is usually described as a rigid backbone in the composite anodes, which resists mechanical stress and morphological degradation. Nevertheless, for samples with a coarse microstructure, a significant decrease of 25% in the size of bulges is observed upon redox cycling (Figure 6d). In the fine and medium samples the changes in the size of the bulges (*r*_50,max_) are small (–6% for fine and +4% for medium). In YSZ the bottleneck radii change considerably upon redox cycling. For example, in the coarse sample the initially small bottleneck radius (*r*_50,min_) decreases again by 90%. However, in fine and medium samples the neck radii decrease strongly (Figure 6e).

The average sizes of bulges and bottlenecks in the porous network, as well as the corresponding constriction factors, are shown in Figure 6g–i. The size of the bulges and the bottlenecks of the pores increase as the initial microstructures become coarser. The biggest change in the pore structure upon redox cycling concerns the increase of the bottleneck radius in fine anodes (Figure 6h), which may be related to the qualitatively observed swelling. Before redox cycling, the constriction factor (β_Pore_) in the fine sample is relatively low (0.25), compared to medium and coarse samples. After redox cycling the β_Pore_ is relatively large (0.5 ± 0.1) in all three samples.

### 3.5. Geodesic Tortuosities (τ)

**Figure 7:**
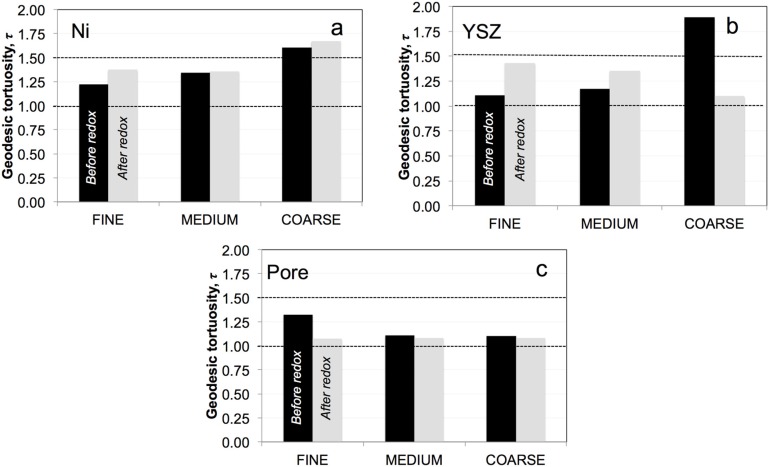
Mean geodesic tortuosity of (**a**) Ni; (**b**) YSZ; and (**c**) pores before (black) and after (gray) redox cycling. Note: Geodesic tortuosities are usually relatively low (*i.e.*, <1.5) and represent the shortest pathways from A to B (in a predetermined direction) through the voxel-domain of a segmented phase.

The geodesic tortuosities of Ni, YSZ, and the pores are shown in Figure 7. In general, the geodesic tortuosities vary only in a narrow range between 1.0 and 1.5. Exceptionally high tortuosities are observed in the coarse samples for Ni (1.7 before and 1.6 after redox) and for YSZ (before redox). Before redox cycling, the tortuosities of Ni and YSZ tend to increase from fine to coarse (Figure 7a,b). Upon redox cycling, the tortuosities of Ni and YSZ tend to increase in all samples except for YSZ in the coarse anode. However, the results for YSZ of the coarse sample must be questioned because the representative elementary volume (REV) may be larger than the image window. The REV for coarse YSZ is very large due to the significant loss of percolation upon redox cycling (see also Figure 5).

In the initial state, the tortuosities in the pore structures (Figure 7c) increase from coarse to fine samples, which may be attributed to stronger densification of the fine anode upon sintering. After redox cycling, the tortuosities in the pores are generally low, which is due to the macroscopic swelling and associated opening of the pore networks.

### 3.6. Simulation of Effective Transport Properties (σ_eff_) and Microstructure Factors (M_sim_)

Effective conductivities and the corresponding microstructure factors (*M*_sim_) can be assessed by means of numerical simulations. In order to capture the microstructure effects in a realistic way, 3D information from tomography is used as geometric input. In this study, the transport simulations are performed with the commercial software GeoDict.

**Figure 8:**
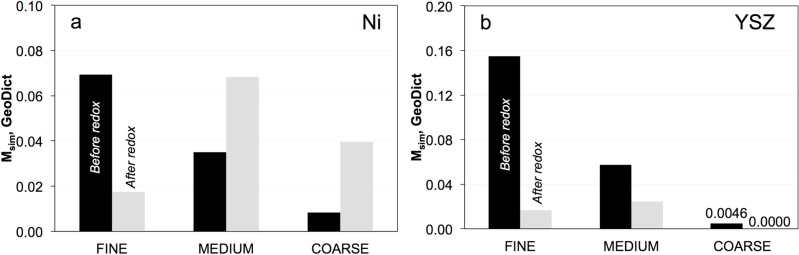
Simulated M-factors (*M*_sim_) of (**a**) Ni and (**b**) YSZ before (black) and after (gray) redox cycling for fine, medium, and coarse samples. Pristine samples exhibit a decreasing *M*_sim_ from fine to coarse for Ni and YSZ. After exposure to redox cycling, the *M*_sim_ of Ni in the fine sample decreases, while it improves in medium and coarse samples. *M*_sim_ of YSZ in all samples decreases after redox cycling.

As shown in Figure 8b, the M-factors (*M*_sim_) of YSZ in the fresh samples tend to decrease from fine to coarse. Hence, fine microstructures appear to be preferable. However, the M-factors of YSZ decrease strongly upon redox cycling, which leads to strong limitations for ionic conductivities. In the coarse microstructure this limitation is already present in the initial microstructure (before redox) due to the low sintering activity and strong constrictions at the bottlenecks.

The trends observed for ionic conductivity in the simulation (*i.e.*, decreasing M-factors from fine to coarse) are caused mainly by the reduction of bottleneck radii for coarser microstructures and by the associated reduction of constriction factors. These variations can be attributed to the inverse relationship between the particle size of YSZ and the corresponding sintering activities (*i.e.*, coarser YSZ tends to have smaller bottlenecks). Apparently, in the coarse sample the necks are so weak that mechanical stresses from redox cycling even lead to a loss of connectivity between the YSZ particles (see Figure 5: drop of percolation factor) and to a reduction of the effective volume fraction. In the fine sample there is also an increase of tortuosity, which contributes to the significant reduction of the M-factor during redox cycling.

The M-factors (*M*_sim_) of Ni vary in a relatively narrow range between 0.01 and 0.08 (Figure 8a). Before redox cycling, the M-factors of Ni decrease from fine to coarse. This result implies that in the fresh anode, fine microstructures are preferred due to the relatively high initial effective electrical conductivity. After redox cycling, the M-factor of Ni in the fine sample decreases, while the M-factors of medium and coarse samples increase. Overall, the simulated effective electrical conductivities in all investigated anodes are still relatively high, even after redox cycling, since they reach 1% to 8% of the intrinsic conductivity in bulk nickel. As mentioned above, since transport distances in thin anode layers are short, these effective electrical conductivities are not expected to impose significant limitations to the anode performance. However, as discussed later, the results for Ni in the medium and coarse microstructure have to be interpreted carefully, because the corresponding REV is very large, possibly even exceeding the thickness of the anode layer.

## 4. Discussion

This study focuses on three main points. (1) The first is the evaluation of the possibility to predict anode conductivities based on an empirical relation (Equation (4)) between the measured topological parameters and effective transport properties. The empirical equation was developed by means of virtual materials testing (*i.e.*, analyzing virtual microstructures from a stochastic model). In this first part, the aim is to test whether this relationship also holds for real microstructures (*i.e.*, porous Ni-YSZ anodes); (2) Next is the understanding of redox degradation mechanisms in Ni-YSZ anodes, with a special focus on effective transport properties. The knowledge of micro-macro relationships (Equation (4)) enables us to determine specific contributions to the degradation of conductivities, which originate from quantifiable changes of specific, transport-relevant topological parameters. This level of detail is novel and potentially sheds new light on degradation mechanisms; (3) The last point is to formulate fabrication guidelines and design concepts for improved redox-stable Ni-YSZ anodes. The mechanisms and degree of degradation are related to the initial microstructure (fine, medium, coarse). This information can be used to test new concepts for redox-stable anode microstructures with high electrical and ionic conductivities, which may be obtained by mixing powders with different levels of fineness.

### 4.1. Prediction of Effective Transport Properties

In the results section, we have presented topological parameters that are relevant for transport properties. In addition, the effective electrical and ionic conductivities were determined by numerical simulation (*M*_sim_) using 3D microstructures as input. At this point we intend to predict the effective electrical conductivity based on the topological parameters using Equation (4). A plug-in of the topological parameters (Φ_eff_, β, τ) obtained from 3D microstructure analysis into this equation gives the predicted M-factors (*M*_pred_).

The prediction of effective transport properties and the corresponding electrochemical performance requires a clear definition of the topological parameters that are used for the prediction. For example, in literature, tortuosity factors are measured in several ways. Iwai *et al.* [54], Kishimoto *et al.* [55], and Ananyev *et al.* [56] used the random walk method, Wilson *et al.* [13], Izzo *et al.* [22], and Laurencin *et al*. [57] used the solution to the Laplace equation, Iwai *et al.* [54] and Grew *et al.* [23] used the Lattice Boltzmann method, while recent works of Boiguez-Muñoz *et al.* [58] and Brus *et al.* [59] used the modified Stefan-Maxwell model. The differences in the definition and implementation of these methods can be one source for the discrepancies between the predicted properties and the experimentally determined properties. A second problem is related to the representative elementary volume (REV), which may also affect the reliability of measured topological parameters. For example, the work of Kanno *et al.* [60] showed that when compared to other microstructural parameters such as volume fraction and surface, the tortuosity factor requires a larger sample volume for representative analysis [61]. A third problem for the prediction of effective properties based on topological parameters is the fact that, often, not all relevant microstructure effects are captured. Very often only tortuosity and effective volume are considered, whereas the bottleneck effect (*i.e.*, constrictivity) is neglected. Despite these challenges, recent studies [58,61,62] show that it is possible to extend qualitative correlation between topological parameters and electrochemical performance to the simulation and prediction not only of effective transport properties but also of electrochemical performance.

While this work considers the microstructure influence on effective transport properties based on quantitative 3D analysis, other works in literature on porous media [63,64] base the transport-related parameters on a solution of transport equations involved to describe conductance [63] or permeability [61]. Similar to this work, effective volume fraction, tortuosity, and constrictivity are identified as the three main parameters that describe the microstructure influence on effective transport properties.

**Figure 9:**
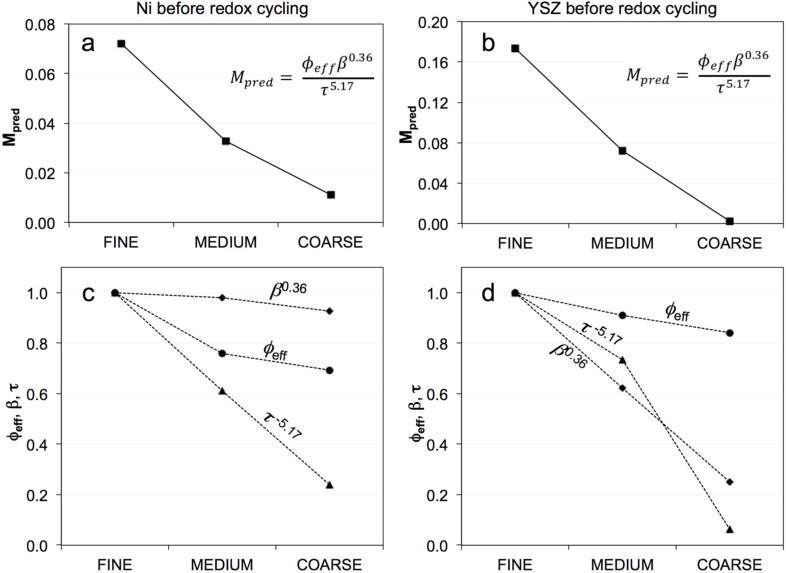
(**a**,**b**) Summary of *M*_pred_ (≈*M*_sim_) and (**c**,**d**) topological parameters in pristine samples of fine, medium, and coarse anodes. The M-factor of Ni is significantly affected by tortuosity, while the M-factor of YSZ is strongly affected by both tortuosity and constrictivity.

A summary of the topological parameters for Ni and YSZ, as well as the predicted M-factors from Equation (4), are listed in Table S4 in the supplementary materials. As shown in Figure 9, the *M*_pred_ of Ni and YSZ before redox cycling follows the same trends as *M*_sim_ (Figure 8). The trends show the highest effective conductivities for the fine anode, which decrease to the coarse anode. Figure 9c,d also present the specific contributions of each topological parameter weighted by the exponents from the empirical Equation (4) (*i.e.*, Φ_eff_, β^0.36^, τ^−5.17^) and normalized against the values of the fine anode. The decrease of *M*_pred_ for Ni from fine to coarse is highly influenced by changes of the tortuosity and effective volume fraction, while the decrease of *M*_pred_ for YSZ is related to variations in constrictivity and tortuosity. The variation of constrictivity in YSZ is compatible with particle size-dependent sintering activity, which was discussed earlier (see Figure 6).

**Figure 10:**
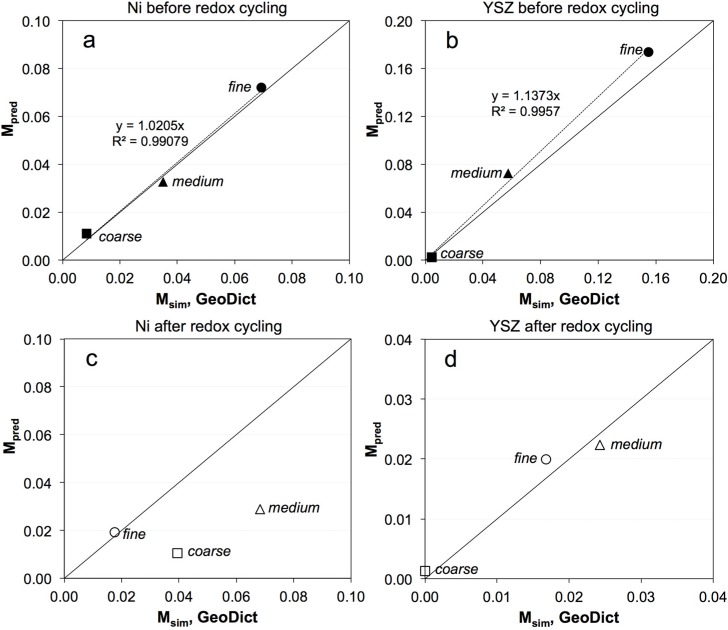
Comparison between predicted (*M*_pred_) and simulated (*M*_sim_) microstructure factors of Ni and YSZ in pristine anodes (**a**,**b**) and after redox cycling (**c**,**d**). Before redox cycling, the data points lie close to the diagonal reference line (solid line), which confirms a strong correlation between the *M*_pred_ and *M*_sim_. (**c**) After redox cycling, the data points for the Ni in the fine anode lie close to the reference line, whereas the data points of medium and coarse anodes are clearly off the diagonal reference line (weak correlation between the *M*_pred_ and *M*_sim_).

The correlations between *M*_pred_ and *M*_sim_ are shown in Figure 10. Before redox cycling, a strong correlation between *M*_pred_ and *M*_sim_ is observed (Figure 10a,b). This is a clear indication that the empirical relationship in Equation (4) is indeed able to predict the effective transport properties of real microstructures. However, for samples after redox cycling (Figure 10c,d) the correlation plot shows a more complex pattern. A good correlation between *M*_pred_ and *M*_sim_ is only obtained after redox cycling for YSZ (Figure 10d). However, for Ni, it is only for the fine anode as shown in Figure 10c. The data points of medium and coarse anodes plot at some distance away from the reference line. The reason for this mismatch is not clear. However, it can be suspected that it may be related to greater heterogeneity in the microstructure, which leads to larger REV, as it is shown in the following. If the REV becomes similar in size or larger than the sample, our analysis becomes flawed, which is suspected to be the case of the medium and coarse microstructure after redox cycling.

**Figure 11:**
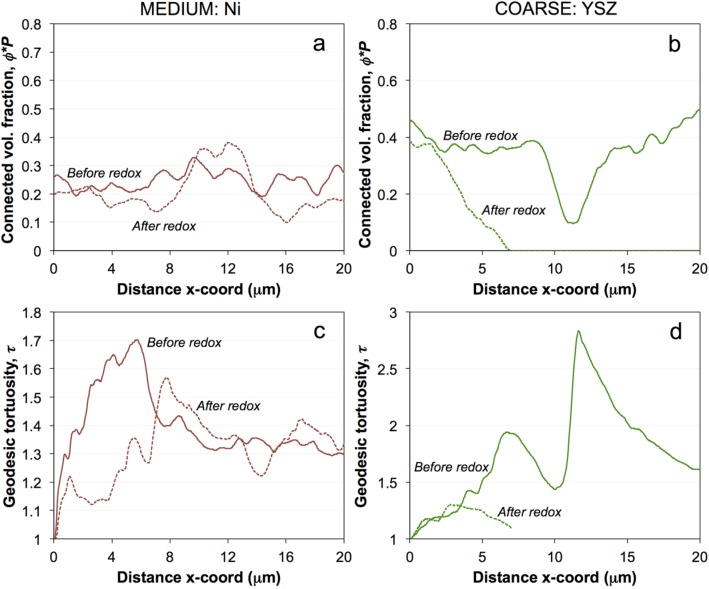
(**a**,**b**) Profiles of the connected volume fraction (Φ*P* = Φ_eff_) and (**c**,**d**) tortuosity (τ) of Ni and YSZ in medium and coarse samples, respectively, as a function of film thickness (*x*-direction). Connectivity check is performed with the inlet plane (left side). The fluctuating values for YSZ are attributed to significant loss of connectivity, which is particularly strong after redox cycling (Compare Figure 5). Geodesic tortuosities are more fluctuating (values between 1 and 2.5) as compared to the fine sample (Figure S2).

The different degrees of heterogeneity and associated potential problems with the size of the REV are illustrated in Figure 11 (medium and coarse). The figures show fluctuations of two topological parameter profiles through the anode layers. Figure 11a,b represent the effective volume fractions from stacked 2D analysis, whereby for each 2D image (*y*–*z* plane) a connectivity check is performed with the inlet plane on the left (x-direction). If connectivity with the left side is lost completely, then the effective volume fraction drops to zero. Minor fluctuations of the volume fractions may arise when the size of 2D slices is not fully representative. Figure 11c,d show the fluctuations in tortuosity. Here each value at a specific distance (*x*) includes a complete 3D analysis (*i.e.*, transport pathways from location *x* to the inlet plane are measured). Close to the inlet plane, the pathways typically have straight connections, which results in tortuosity values close to one. A plateau value is reached at larger distances, when the thickness is large enough to include a representative volume (e.g., see Figure S2 in the supplementary materials for the volume fraction and tortuosity profiles of the fine anode).

For the medium and coarse anode, the fluctuations of volume fractions are high (Figure 11a,b). In particular, for coarse YSZ after redox cycling, the effective volume fraction drops to zero at a distance of *ca.* 7 μm, which is attributed to a loss of connectivity. However, the apparently complete loss of connectivity might be related to a limited size of the image window. Some connected pathways may potentially exist outside the image window. For tortuosity (coarse anode), the fluctuations are even higher. The curve for YSZ after redox cycling also ends at a distance of *ca.* 7 μm, which is again due to loss of connectivity (Figure 11d). Overall, the coarse anode has a much larger REV that is not always captured in the image windows. Especially for the coarse anode after redox cycling, the size of the tomographic data is smaller than the REV, which may explain the mismatch between *M*_pred_ and *M*_sim_ in the correlation plot (Figure 10c,d). Similar problems are identified for the medium-grained anode after redox cycling.

**Figure 12:**
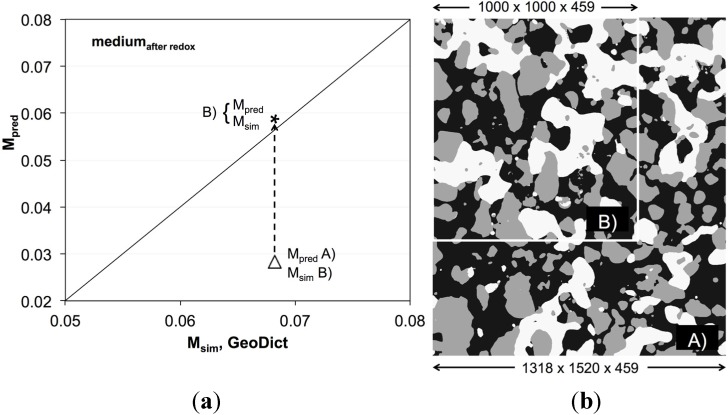
(**a**) Comparison between *M*_pred_ and *M*_sim_ for Ni, illustrating the effect of varying image window size. The point represented by a star (*) is obtained if both *M*_pred_ and *M*_sim_ are obtained by analysis of the same image window (region B, (**b**) image). The data point marked with an open triangle (Δ) is obtained if *M*_pred_ is based on analysis of region A (**b**) while *M*_sim_ is obtained from region B (**b**). Note: The image on (**b**) is a 2D cross-section of a 3D image window. The larger image window (A, in 3D) exceeds the limits of conventional voxel-based computations (*M*_sim_) but can be used for topology analysis (*M*_pred_).

In the context of concerns over the REV size, it must be emphasized that computational limitations are generally higher for the numerical simulation compared to the ones for topology analysis. Due to the limitation in memory and computing time, smaller sub-volumes are used as a structural input for numerical simulations with GeoDict. Figure 12 represents a correlation plot of the medium-grained anode, which highlights the problems related to different image window sizes used for simulation and image analysis. Before redox cycling, a reasonable correlation is obtained between *M*_sim_ and *M*_pred_ (Figure 10a), although the simulation is performed on the basis of a smaller 3D image window. Hence, even with the smaller image window, a representative analysis is obtained for pristine anodes. Upon redox cycling, the heterogeneity increases and, thus, the REV becomes larger. The corresponding data point represented by an open triangle (Figure 12a) is far away from the reference line. In this case the topology analysis is based on a larger image window (marked with “A” in Figure 12b) compared to the one for the numerical simulation (marked with “B”). The size dependence indicates that at least with the smaller window “B”, the REV is not reached. If both analyses are based on the same image window, then the correlation is still good (see data point marked with a star in Figure 12a). Hence, even if the image window is below REV (as it is in this case), a good correlation can be achieved. This finding can be considered as an additional confirmation that the empirical relationship (Equation (4)) is capable of reliably predicting the effective transport properties. On the other side, it must be noted that throughout this study, numerical simulations are performed on smaller image windows than the corresponding topology analyses. Good correlations of *M*_sim_ and *M*_pred_ (e.g., for all anodes before redox cycling) thus indicate that these analyses are based on a representative image window.

### 4.2. Degradation Mechanism

Effective conductivities from the simulation (*M*_sim_) and from the image analysis (*M*_pred_) show a good correlation, provided REV is not too large with respect to the sampled image size. This is interpreted as a positive validation of the underlying empirical relationship (Equation (4)). However, both the simulation and image analysis basically use the same type of data from the tomography as input. A thorough validation should therefore be based on independent measurements. Hence, the experimental electrical conductivities presented in Figure 3 can be used for this.

**Figure 13:**
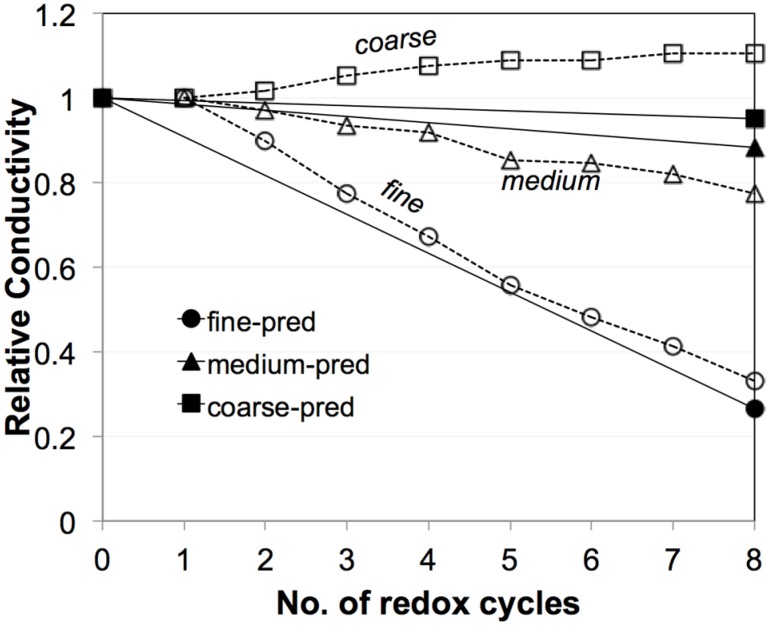
Relative experimental conductivities (open symbols) for fine, medium, and coarse anodes and their evolution over eight redox cycles at 950 °C based on the work of Iwanschitz [45]. Relative experimental conductivity means that the measured conductivity after *n* cycles is normalized by the conductivity measured after the first cycle. The relative predicted conductivity (filled symbols) is based on the *M*_pred_, with normalization by the initial values (zeroth cycle). Similar evolution curves are obtained by prediction and experiment.

Figure 13 shows a comparison of experimental data (open symbols) and *M*_pred_ from the image analysis (filled symbols), and it shows how these properties degrade upon redox cycling. The experimental data are presented as relative conductivity, whereby for each data series the measured electrical conductivity after *n* redox cycles is normalized by the measured conductivity after the first redox cycle for the considered series. As discussed earlier, the experimental data (Figure 3) shows linear trends between the first to eighth redox cycles, which is interpreted as a result from microstructure degradation. However, the initial values (zeroth) and the degradation during the first cycle also show strong irregularities for the three different anodes, which are speculated to result from experimental artifacts. The influence of these uncertainties can be suppressed by taking the value after the first cycle (instead of the zeroth) as a basis for normalization.

Normalized M-factors are based on tomographs from anodes after the zeroth and eighth cycle. In the tomographic data, the 3D microstructure after the first cycle was not obtained. Nonetheless, the comparison shows that the trends for degradation upon redox cycling are very similar for experimental conductivities and for the predicted M-factors. In both cases, the fine anodes show a high degradation rate, whereas degradation rates in the medium- and coarse-grained anodes are very low. For the coarse anode the experimental measurements even indicate a positive effect of redox cycling (*i.e.*, increasing conductivities after redox exposure). In summary, the experimental results support the validity of the suggested empirical relationship (Equation (4)) to predict effective conductivity. Even though for some samples (e.g., coarse, after redox) the image size of the tomographs is at the lower REV limit, there is still a good correlation between predicted and experimentally measured degradation rates.

**Figure 14:**
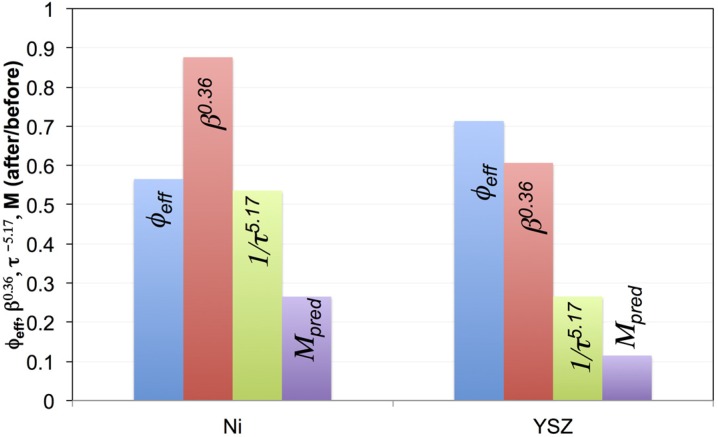
Redox degradation of the fine anode is described with normalized M-factors and topological parameters of Ni and YSZ. Normalization means building the ratio of values before over values after redox cycling. The degradation of the Ni M-factor is significantly affected by changes in volume fraction (*i.e.*, percolation) and tortuosity, whereas the degradation of the YSZ M-factor is most significantly affected by changes in tortuosity. The exponents of the microstructural parameters are those from Equation (4), which allows us to directly assess the relative roles of these terms on the M-factors.

**Figure 15:**
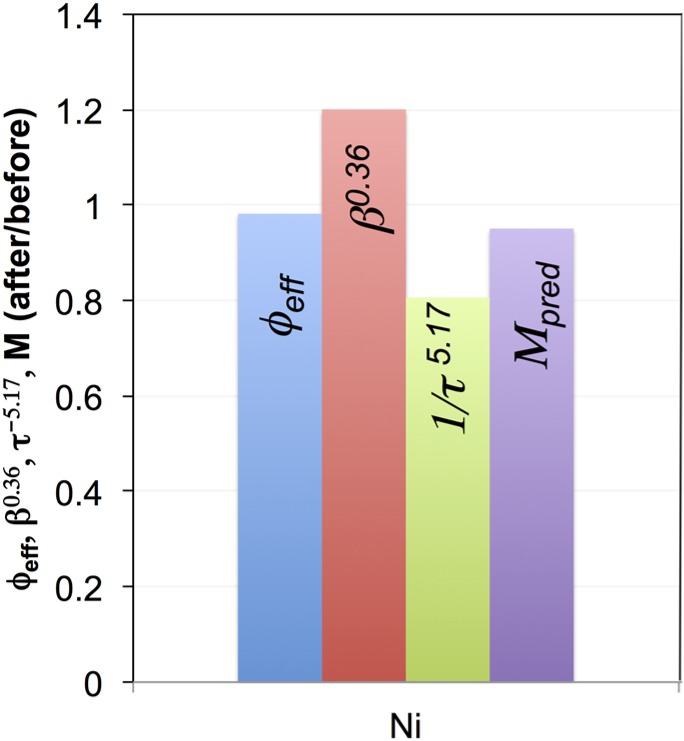
Illustration of redox degradation by normalized M-factors and topological parameters for Ni in the coarse anode (*i.e.*, ratio of values before/after redox). Upon redox cycling, the M-factor (Ni, coarse) and associated electrical conductivity do not degrade since the effect of worsened tortuosity (normalized τ^−5.17^ < 1) is compensated by improved constrictivity (normalized β^0.36^ > 1). The exponents of the microstructural parameters are those from Equation (4), which allows us to directly assess the relative roles of these terms on the M-factors.

In the following section, the modes of degradation after redox cycling are discussed separately for the fine and coarse samples. In Figure 14 and Figure 15, the normalized M-factors and normalized topological parameters are used in order to illustrate details of microstructure degradation. Normalization in this case means plotting the ratios of values obtained after eight redox cycles over the values from pristine samples. The ratio of the microstructural parameter is elevated to the same power as in Equation (4) so that this ratio directly reflects the impact on the M-factor change.

This reveals that in the fine sample, the degradation of the normalized M-factor of Ni is mainly due to changes of tortuosity and volume fraction. This is compatible with results in Figure 4 and Figure 7 where a significant decrease in connectivity (effective volume fraction) and an increase in tortuosity are observed in the fine sample. For YSZ, the normalized M-factor of only 0.1 indicates a strong degradation. This is caused by significant changes in tortuosity, and to a lower degree, by changes in constrictivity. It is important to note that Ni agglomeration is a very evident feature in all Ni-based anodes. However, with respect to the degradation of effective transport properties, our results for the fine anode indicate that the degradation rates are higher for YSZ (*i.e.*, ionic conductivity) than for Ni (*i.e.*, electrical conductivity). This is surprising, considering that a relatively high sintering activity in fine microstructures leads to relatively strong bottlenecks of YSZ. This example thus also illustrates that detailed quantitative analysis can provide counterintuitive results which contradict widespread interpretations of Ni agglomeration that are mainly based on qualitative observations that suffer from lack of microstructural characterization and quantification.

For the coarse sample, the influence of the topological parameters on the degradation of *M*_pred_ in Ni is shown in Figure 15. In general, the transport properties of Ni do not degrade during the eight redox cycles because the influence of a worsened tortuosity is compensated by improved constrictivity (*i.e.*, wider bottlenecks). In addition, unlike for the fine anode, redox cycling did not involve remarkable loss of connectivity in the fine Ni network. For YSZ in coarse anodes, the redox cycling leads to significant degradation of the M-factor and the associated ionic conductivity. This is attributed to the substantial loss of connectivity (see Figure 5 and discussion of size-dependent sintering activity). This loss of connectivity also leads to a larger REV, which questions the reliability of the corresponding topological analyses of coarse YSZ after redox cycling.

**Figure 16:**
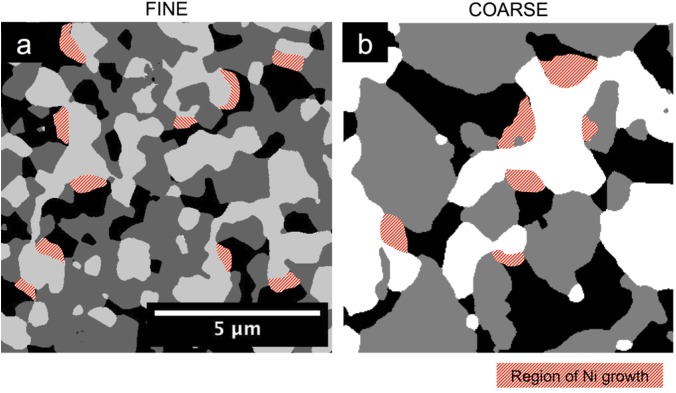
Different modes of Ni agglomeration and growth in Ni-YSZ anodes during redox cycling. In the fine anode (**a**) where particles are more granular, Ni growth is mainly located around the Ni bulges. In the coarser anode (**b**), Ni forms irregularly shaped domains (not round particles), and growth is often located at the bottlenecks (*i.e.*, at concavities with relatively high surface energy).

Our interpretation of microstructural results leads to the postulation of different modes of Ni growth, depending on the coarseness of the initial microstructure. These modes are qualitatively illustrated in Figure 16. For the fine sample, Ni coarsening happens mainly along the bulges of the Ni network (indicated as red domains in Figure 16a). It is suggested that the smallest Ni particles from the initial microstructure have a relatively high mobility due to a high surface energy. Preferably, the mobile Ni becomes enriched at the pre-existing bulges in the Ni network and/or they may also form new isolated islands. This is also supported by the results for the fine anode in Figure 6, which show that the size of the Ni bulges (*r*_50,max_) increases during redox cycling and the connectivity of Ni decreases at the same time. In addition, in the fine microstructure, YSZ and pores both form fine-structured networks which do not leave much space for unconstrained Ni coarsening. As a consequence, the reorganization of Ni also induces strain in the YSZ phase which is localized along the bottlenecks. This also leads to lower constrictivity in YSZ and to lower predicted ionic conductivity.

For coarse anode microstructures, the Ni growth is mainly localized along the bottlenecks (as indicated by red domains in Figure 16b). As shown in Figure 6, for coarser samples, the increase in constrictivity is mainly caused by an increase in bottleneck sizes. These results explain why the electrical conductivity and associated M-factors for Ni are more stable in coarse than in fine anodes. According to experimental results, redox cycling may even lead to higher electrical conductivities, which may be attributed to the widening of bottlenecks.

**Figure 17:**
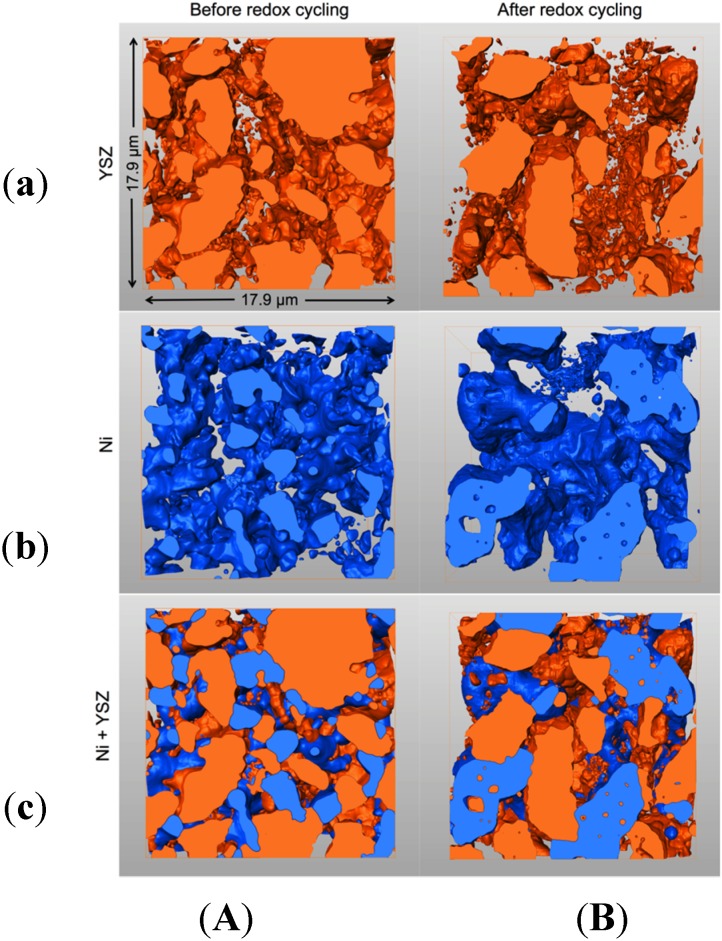
3D visualization of YSZ (**a**); Ni (**b**); and Ni + YSZ (**c**) in coarse sample before (**A**) and after (**B**) redox cycling. Redox degradation invokes a splitting of YSZ particles and disintegration into numerous disconnected islands (**a**). Ni agglomeration leads to the formation of a less granular structure (**b**). Ni is partially overgrowing the small, disconnected YSZ islands (**c**).

While Ni coarsening is an obvious feature of redox degradation, YSZ is also affected by the stress associated with the Ni agglomeration. For example, Figure 17 shows the splitting of YSZ particles invoked by the Ni growth. The strain is most probably concentrated along the sinter necks, which are weakest for the coarse anode. Consequently, the YSZ disintegrates and loses percolation.

In summary, the different modes of Ni agglomeration can be explained by the fact that the fine microstructure has a granular texture with numerous fine particles. The reduction of surface energy is achieved when Ni is redistributed from small particles to larger ones. In contrast, in the coarse microstructure, Ni forms a more continuous network with a sinusoidal surface morphology. The surface energy is minimized when mobile Ni moves into the concavities (*i.e.*, bottlenecks).

### 4.3. Mixing Different Powders

The optimization of redox stability of Ni-YSZ has also been investigated by using various techniques such as the addition of Mg species [65], the infiltration of a polymeric NiO precursor into the YSZ skeleton [66], the dispersal of fine YSZ particles on the surface of NiO [67], and the lowering of the reoxidation temperature [68].

Based on the knowledge gained in this study, we suggest addressing the issue of redox degradation by combining the advantages of different microstructure fineness (see Table 1). According to this study, the fine anode has the highest initial electrical and predicted ionic conductivities. Moreover, it has the advantage of strong YSZ bottlenecks, which ensure relatively stable ionic conductivity (and, supposedly, high TPB). However, in terms of redox stability, the anode with coarse microstructure has distinct advantages, mainly because it has a higher initial porosity which leaves more space for the volume expansion upon oxidation. In the coarse anode, Ni undergoes a low loss of percolation and the mobilization of Ni even leads to wider bottlenecks, which have a positive effect on electrical conductivity. The disadvantage here is the relatively low sintering activity of YSZ. Consequently, for improved redox stability, we combine a 50:50 vol % ratio of fine and coarse, as well as fine and medium, powders of YSZ. This should lead to higher initial conductivities (from fine) and increase the redox stability of Ni (coarser pores) and of YSZ (fine particles as sinter help).

**Table 1:** Summary of degradation in Ni and YSZ upon redox cycling of fine and coarse anodes.

State of Degradation	FINE		COARSE	
	Ni	YSZ	Ni	YSZ
Before redox cycling	(+) High initial e^−^ conductivity	(+) High initial ionic conductivity	(–) Low initial e^−^ conductivity	(–) Low initial ionic conductivity
After redox cycling	(–) Strong coarsening, (–) Loss of percolation, (–) Increase of τ	(+) Strong bottlenecks due to high sinter activity, (+) Small increase of τ	(–) Significant coarsening but, (+) Low loss of percolation, and (+) Low change of β and τ	(–) Weak bottlenecks due to low sinter activity, (–) High loss of percolation, (−) Strong change of β and τ

**Figure 18:**
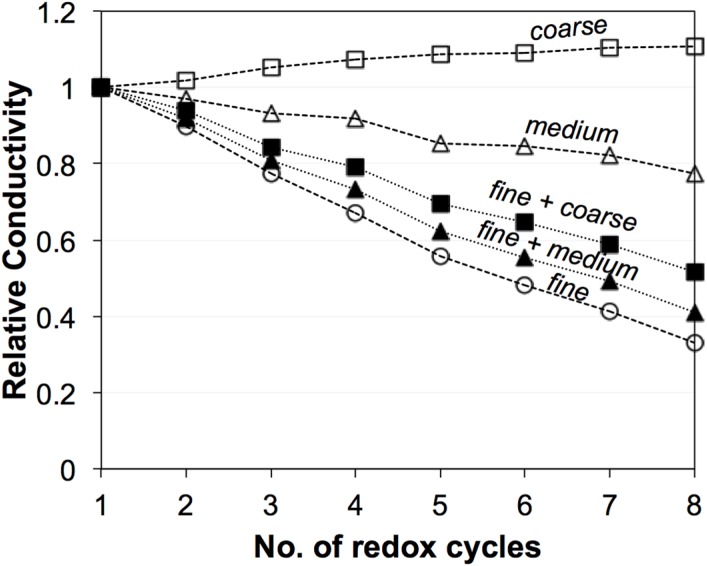
Relative conductivities of anodes fabricated with mixed powders (*i.e.*, 50%/50% fine-medium and 50%/50% fine-coarse, respectively) compared to fine, medium, and coarse [45]. The “mixed anodes” show a similar behavior as the fine anode, which indicates that the fraction of fine powders is too high.

The relative electrical conductivities (Figure 18) show that in 50:50 mixtures the degradation behavior is mainly dominated by the properties of the fine powder and microstructure. The addition of medium and coarse powders seems to lower the degradation rate of the electrical conductivity but, obviously, the 50% fine is too high. For further optimization we thus suggest a lower amount of the fine powder (e.g., 10%). Such anode compositions should have better resistance to both ionic and electrical conductivity degradation.

It must be emphasized that in this study we focused on the effective transport properties. Therefore, a strong limitation is the lack of experimental measurements for ionic conductivity. Since the intrinsic conductivity of YSZ is much lower than the one of Ni, it follows that with respect to anode performance, the effective ionic conductivity is also more limiting than the electronic conductivity [69]. These investigations show that degradation of YSZ (*i.e.*, reduction of M-factor) during the redox cycling can be even worse than the degradation of Ni. Significant loss of connectivity is observed for coarse YSZ, which hinders the distribution of ions throughout the anode layer. In principle, the properties of an anode do not have to be constant over the entire thickness. The functional layer close to the anode-electrolyte interface should have high electrochemical activity (TPB length) and high ionic conductivity. The anode regions at larger distances away from this interface serve as current collectors and, hence, they should have high electrical conductivity. This leads to the well-known concept of double-layered anodes [20,24,25]. A fine-grained anode is used as the functional layer (e.g., with 60 vol % YSZ *vs.* 40 vol % Ni) and the coarse-grained material is suitable to serve as a current collector (e.g., with 50 vol % YSZ *vs.* 50 vol % Ni). In this context, the addition of small amounts of fine YSZ (e.g., 5 vol %) to a generally coarse anode microstructure may lead to a current collector with stronger bottlenecks. However this latter hypothesis has not yet been tested.

## 5. Conclusions

In this study, we investigate the relationship between microstructure and effective transport properties in Ni-YSZ anodes and the corresponding degradation upon redox cycling. Our microstructure investigations reveal a complex pattern of different materials properties and degradation behaviors, which vary depending on the coarseness of the initial microstructure.

### 5.1. Comparison of Fine *vs.* Coarse Anodes: Before Redox Cycling

In the initial state, the fine anode has higher-predicted electrical and ionic conductivities compared to the coarse anode. The M-factors predicted from the topology analysis and from the numerical simulation are consistent with each other. These M-factors decrease from fine to coarse microstructures for both phases (Ni and YSZ). For Ni, the lower M-factor in the coarse anode is mainly related to a higher tortuosity and a lower effective volume fraction (due to low connectivity). For YSZ, the lower M-factor in the coarse anode can be attributed to a higher tortuosity and a lower constrictivity (small bottlenecks, low sinter activity).

### 5.2. Comparison of Ni in Fine *vs.* Coarse Anodes: After Eight Redox Cycles

Upon redox cycling at 950 °C, Ni coarsening and YSZ loss of percolation are the most obvious features of anode degradation, but the degree of degradation strongly depends on the initial particle size and on the fineness of the microstructure. The fine anode is affected by substantial Ni coarsening, which leads to a significant loss of percolation and increase of tortuosity. This microstructure degradation is compatible with the experimentally measured drop of electrical conductivity by *ca.* 50% over eight redox cycles. In the coarse microstructure Ni coarsening is also a prominent feature; however, due to larger pores, the spatial rearrangement of Ni is less harmful than in the fine anode. In the coarse anode, the loss of percolation as well as the changes of tortuosity and constrictivity are relatively small. Moreover, this microstructure degradation is compatible with the experimental data, showing a stable evolution of the electrical conductivity over eight redox cycles.

### 5.3. Comparison of YSZ in Fine *vs.* Coarse Anodes: After Eight Redox Cycles

Redox cycling and the associated rearrangement of Ni exerts stresses onto the rigid YSZ structure. In the fine sample, YSZ has stronger necks due to a higher sintering activity. Consequently, the fine YSZ remains relatively stable upon redox cycling. In contrast, the coarse YSZ has a lower mechanical stability due to weaker sinter necks. In coarse anodes, redox cycling leads to a significant loss of connectivity between the large YSZ grains despite the fact that the pores are more open and Ni has more room for volume expansion upon oxidation. The loss of connectivity also induces a strong increase of tortuosity. Together with a decrease in constrictivity, these three degradation phenomena (*i.e.*, changes of percolation *P*, tortuosity τ, and constrictivity β) lead to a significant drop of the YSZ M-factor and of the corresponding predicted ionic conductivity in the coarse anode.

Also, in this study, we followed a hypothesis that a mixture of fine- and coarse-grained (or medium-grained) particles would be beneficial to slow down the degradation of both phases (Ni and YSZ) and the associated drop of conductivities. The first experimental results in this direction indicate that mixtures with 50% fine and 50% coarse YSZ powders are not ideal. In the 50:50 (fine:coarse) mixtures the Ni coarsening and YSZ degradation appear to be dominated by the negative properties of fine particles and pores. Consequently, it is suggested that a smaller amount of fine YSZ may be sufficient to increase the stability of the sinter necks between larger YSZ particles. This improvement is important since, in general, ionic conductivity is a strongly limiting factor for high anode performances (*i.e.*, distribution of O^2−^ ions within the anode). In addition, relatively coarse pores are considered to be beneficial in the sense that they represent buffer space during the expansion upon Ni oxidation to NiO, which is not available in the fine-grained anodes due to the higher density after sintering. The coarser pores may also be introduced with suitable pore formers. In conclusion, for future improvements of anode performance and redox stability, we propose a mixture of (<50%) fine and (>50%) coarse YSZ powders and the addition of relatively coarse pore formers. The optimum proportions of these components are yet to be defined.

Aside from the insights mentioned above, the following conclusions can be drawn with respect to the suitability of methods to study microstructure effects. In this study, the relationship between conductivity and microstructure was investigated in two ways: (1) by numerical simulation of conductivity and (2) by using an empirical relationship that allows the prediction of effective conductivities based on topological parameters from 3D analysis. With respect to numerical simulation it is important to note that, in the past, conventional FE (finite element) analysis was strongly limited by the number of structural elements (grid points). Therefore, it was necessary to transform the high resolution tomographic data (voxels) into lower resolution meshes (cubic or tetragonal). However, recent progress of commercial simulation tools (e.g., GeoDict, Avizo) allows running simulations on voxel grids > 500^3^ in a reasonable time. The option to perform computations directly on the voxel grid from tomography represents a considerable improvement. For the initial anode microstructures investigated in this study, this size is sufficient to describe the REV at a resolution that is high enough to capture the relevant details (e.g., small bottlenecks and connectivity). However, after redox cycling, the representative volume is clearly larger and the results obtained from the numerical simulation are not reliable. The second method (*i.e.*, topological analysis) can handle larger data volumes and the limitations regarding REV are less severe. Nevertheless, for the coarse anode after redox cycling, the structures are very heterogeneous and the REV is strongly enlarged. This size could not be reached with either of the methods. For this reason, the results for coarse anodes after redox cycling must be considered as qualitative and preliminary.

A further methodic aspect is the fact that transport simulations do not provide the specific topological parameters which control the anode performance. 3D imaging and topological analyses are necessary to capture the complex picture of microstructure degradation and its relation with effective properties. In summary, since both methods provide complementary information, a combination of the two methods is considered as being ideal to study such microstructure effects.

## Supplementary Materials

Supplementary materials can be accessed at: http://www.mdpi.com/1996-1944/8/9/5554/s1.

## References

[1] Gauckler L.J., Beckel D., Buergler B.E., Jud E., Muecke U.P., Prestat M., Rupp J.L.M., Richter J. (2004). Solid Oxide Fuel Cells: Systems and Materials. CHIMIA Int. J. Chem..

[2] Mantzaras J., Freunberger S.A., Büchi F.N., Roos M., Brandstätter W., Prestat M., Gauckler L.J., Andreaus B., Hajbolouri F., Senn S.M. (2004). Fuel cell modeling and simulations. CHIMIA Int. J. Chem..

[3] Adler S.B., Bessler W.G. (2010). Elementary kinetic modeling of solid oxide fuel cell electrode reactions. Handb. Fuel Cells.

[4] Pecho O., Holzer L., Yáng Z., Martynczuk J., Hocker T., Flatt R.J., Prestat M. (2015). Influence of strontium-rich pore-filling phase on the performance of La_0.6_Sr_0.4_CoO_3-δ_ thin-film cathodes. J. Power Sources.

[5] Sarantaridis D., Atkinson A. (2007). Redox cycling of Ni-based solid oxide fuel cell anodes: A review. Fuel Cells.

[6] Klemenso T., Mogensen M. (2007). Ni-YSZ solid oxide fuel cell anode behavior upon reodx cycling based on electrical characterization. J. Am. Ceram. Soc..

[7] Fouquet D., Muller A.C., Weber A., Ivers-Tiffee E. (2003). Kinetics of oxidation and reduction of Ni/YSZ cermets. Ionics.

[8] Iwanschitz B., Holzer L., Mai A., Schütze M. Nickel agglomeration in solid oxide fuel cells under different operating conditions. Proceedings of the 10th European SOFC Forum.

[9] Atkinson A., Barnett S., Gorte R.J., Irvine J.T.S., McEvoy A.J., Mogensen M., Singhal S.C., Vohs J. (2004). Advanced anodes for high-temperature fuel cells. Nat. Mater..

[10] Nelson G.J., Grew K.N., Izzo J.R., Lombardo J.J., Harris W.M., Faes A., Hessler-Wyser A., van Herle J., Wang S., Chu Y.S. (2012). Three-dimensional microstructural changes in the Ni-YSZ solid oxide fuel cell anode during operation. Acta Mater..

[11] Balakrishnan N., Takeuchi T., Nomura K., Kageyama H., Takeda Y. (2004). Aging effect of 8 mol % YSZ ceramics with different microstructures. J. Electrochem. Soc..

[12] Hattori M., Takeda Y., Sakaki Y., Nakanishi A., Ohara S., Mukai K., Lee J.-H., Fukui T. (2004). Effect of aging on conductivity of yttria stabilized zirconia. J. Power Sources.

[13] Wilson J.R., Kobsiriphat W., Mendoza R., Chen H.-Y., Hiller J.M., Miller D.J., Thornton K., Voorhees P.W., Adler S.B., Barnett S.A. (2006). Three-dimensional reconstruction of a solid-oxide fuel-cell anode. Nat. Mater..

[14] Shearing P.R., Cai Q., Golbert J.I., Yufit V., Adjiman C.S., Brandon N.P. (2010). Microstructural analysis of a solid oxide fuel cell anode using focused ion beam techniques coupled with electrochemical simulation. J. Power Sources.

[15] Vivet N., Chupin S., Estrade E., Piquero T., Pommier P.L., Rochais D., Bruneton E. (2011). 3D Microstructural characterization of a solid oxide fuel cell anode reconstructed by focused ion beam tomography. J. Power Sources.

[16] Shikazono N., Kanno D., Matsuzaki K., Teshima H., Sumino S., Kasagi N. (2010). Numerical assessment of SOFC anode polarization based on three-dimensional model microstructure reconstructed from FIB-SEM images. J. Electrochem. Soc..

[17] Holzer L., Muench B., Iwanschitz B., Cantoni M., Hocker T., Graule T. (2011). Quantitative relationships between composition, particle size, triple phase boundary length and surface area in nickel-cermet anodes for Solid Oxide Fuel Cells. J. Power Sources.

[18] Holzer L., Iwanschitz B., Hocker Th., Münch B., Prestat M., Wiedenmann D., Vogt U., Holtappels P., Sfeir J., Mai A., Graule Th. (2011). Microstructure degradation of cermet anodes for solid oxide fuel cells: Quantification of nickel grain growth in dry and in humid atmospheres. J. Power Sources.

[19] Jiao Z., Shikazono N. (2014). Simulation of nickel morphological and crystal structures evolution in solid oxide fuel cell anode using phase field method. J. Electrochem. Soc..

[20] Wilson J.R., Cronin J.S., Barnett S.A. (2011). Linking the microstructure, performance and durability of Ni-yttria-stabilized zirconia solid oxide fuel cell anodes using three-dimensional focused ion beam-scanning electron microscopy imaging. Scr. Mater..

[21] Shearing P.R., Golbert J., Chater R.J., Brandon N.P. (2009). 3D reconstruction of SOFC anodes using a focused ion beam lift-out technique. Chem. Eng. Sci..

[22] Izzo J.R., Joshi A.S., Grew K.N., Chiu W.K.S., Tkachuk A., Wang S.H., Yun W. (2008). Nondestructive reconstruction and analysis of SOFC anodes using X-ray computed tomography at sub-50 nm resolution. J. Electrochem. Soc..

[23] Grew K.N., Chu Y.S., Yi J., Peracchio A.A., Izzo J.R., Hwu Y., de Carlo F., Chiu W.K.S. (2010). Nondestructive nanoscale 3D elemental mapping and analysis of a solid oxide fuel cell anode. J. Electrochem. Soc..

[24] Usseglio-Viretta F., Laurencin J., Delette G., Villanova J., Cloetens P., Leguillon D. (2014). Quantitative microstructure characterization of a Ni/YSZ bi-layer coupled with simulated electrode polarization. J. Power Sources.

[25] Cronin J.S., Chen-Wiegart Y.K., Wang J., Barnett S.A. (2013). Three-dimensional reconstruction and analysis of an entire solid oxide fuel cell by full-field transmission X-ray microscopy. J. Power Sources.

[26] Guan Y., Li W.J., Gong Y.H., Liu G., Zhang X.B., Chen J., Gelb J., Yun W.B., Xiong Y., Tian Y.C., Wang H.Q. (2011). Analysis of the three-dimensional microstructure of a solid-oxide fuel cell anode using nano X-ray tomography. J. Power Sources.

[27] Jang J.H., Ryu J.H., Oh S.M. (2000). Microstructure of Ni/YSZ cermets according to particle size of precursor powders and their anodic performances in SOFC. Ionics.

[28] Ribeiro N.F.P., Souza M.M.V.M., Macedo Neto O.R., Vasconcelos S.M.R., Schmal M. (2009). Investigating the microstructure and catalytic properties of Ni/YSZ cermets as anodes for SOFC applications. Appl. Catal. A.

[29] Guo W., Liu J. (2008). The effect of nickel oxide microstructure on the performance of Ni/YSZ anode-supported SOFCs. Solid State Ionics.

[30] Kawashima T., Miyoshi S., Shibuta Y., Yamaguchi S. (2013). Particle size dependence of polarization of Ni/YSZ cermet anodes for solid oxide fuel cells. J. Power Sources.

[31] Brown M., Primdahl S., Mogensen M. (2000). Structure/Performance relations for Ni/Yttria-Stabilized zirconia anodes for solid oxide fuel cells. J. Electrochem. Soc..

[32] Zhu X., Guan C., Lü Z., Su W. (2013). Effects of initial reduction temperature on the microstructures and performances of Ni/YSZ anodes for solid oxide fuel cells. J. Electrochem. Soc..

[33] Grew K.N., Peracchio A.A., Joshi A.S., Izzo J.R., Chiu W.K.S. (2010). Characterization and analysis methods for the examination of the heterogeneous solid oxide fuel cell electrode microstructure. Part 1: Volumetric measurements of the heterogeneous structure. J. Power Sources.

[34] Gaiselmann G., Neumann M., Schmidt V., Pecho O., Hocker T., Holzer L. (2014). Quantitative relationships between microstructure and effective transport properties based on virtual materials testing. AIChE J..

[35] Wiedenmann D., Keller L., Holzer L., Stojadinović J., Münch B., Suarez L., Fumey B., Hagendorfer H., Brönnimann R., Modregger P. (2013). Three-dimensional pore structure and ion conductivity of porous ceramic diaphragms. AIChE J..

[36] Petersen E.E. (1958). Diffusion in a pore of varying cross section. AIChE J..

[37] Holzer L., Wiedenmann D., Muench B., Keller L., Prestat M., Gasser P., Robertson I., Grobéty B. (2013). The influence of constrictivity on the effective transport properties of porous layers in electrolysis and fuel cells. J. Mater. Sci..

[38] Grew K.N., Peracchio A.A., Chiu W.K.S. (2010). Characterization and analysis methods for the examination of the heterogeneous solid oxide fuel cell electrode microstructure: Part 2. Quantitative measurement of the microstructure and contributions to transport losses. J. Power Sources.

[39] Nelson G.J., Peracchio A.A., Chiu W.K.S. (2011). Analytical investigations of varying cross section microstructures on charge transfer in solid oxide fuel cell electrodes. J. Power Sources.

[40] Nelson G.J., Cassenti B.N., Peracchio A.A., Chiu W.K.S. (2010). Two-dimensional charge transfer and space charge effects in extended surface solid oxide fuel cell electrodes. J. Power Sources.

[41] Clennell M.B. (1997). Tortuosity: A guide through the maze. Geol. Soc..

[42] Stenzel O., Pecho O., Holzer L., Neumann M., Schmidt V. (2015). Predicting effective conductivities based on geometric microstructure characteristics. AIChE J..

[43] Holzer L., Iwanschitz B., Hocker Th., Keller L., Pecho O., Sartoris G., Gasser Ph., Muench B. (2013). Redox cycling of Ni/YSZ anodes for solid oxide fuel cells: Influence of tortuosity, constriction and percolation factors on the effective transport properties. J. Power Sources.

[44] Münch B., Holzer L. (2008). Contradicting geometrical concepts in pore size analysis attained with electron microscopy and mercury intrusion. J. Am. Ceram. Soc..

[45] Iwanschitz B. (2012). Degradation von Ni-Cermet-Anoden in Keramischen *Hochtemperatur Brennstoffzellen*. Ph.D. Thesis.

[46] Iwanschitz B., Sfeir J., Mai A., Schütze M. (2010). Degradation of SOFC anodes upon redox cycling: A comparison between Ni/YSZ and Ni/CGO. J. Electrochem. Soc..

[47] Fiji. http://fiji.sc/Fiji.

[48] Avizo, Version 8.1.1; Software for Scientific and Industrial Data. http://www.fei.com/software/avizo3d/.

[49] Washburn E.W. (1921). The Dynamics of Capillary Flow. Phys. Rev..

[50] Diamond S. (2000). Mercury porosimetry: An inappropriate method for the measurement of pore size distributions in cement-based materials. Cem. Concr. Res..

[51] Thulasiraman K., Swamy M.N.S. (1992). Graphs: Theory and Algorithms.

[52] (2015). GeoDict, Version 2014 Rev; Software for Scientific and Industrial Data. http://www.geodict.com/.

[53] Wiegmann A., Zemitis A. (2006). EJ-HEAT: A Fast Explicit Jump Harmonic Averaging Solver for the Effective Heat Conductivity of Composite Materials.

[54] Iwai H., Shikazono N., Matsui T., Teshima H., Kishimoto M., Kishida R., Hayashi D., Matsuzaki K., Kanno D., Saito M. (2010). Quantification of SOFC anode microstructure based on dual beam FIB-SEM technique. J. Power Sources.

[55] Kishimoto M., Iwai H., Saito M., Yoshida H. (2009). Quantitative evaluation of transport properties of SOFC porous anode by random walk process. ECS Trans..

[56] Ananyev M.V., Bronin D.I., Osinkin D.A., Eremin V.A., Steinberger-Wilckens R., de Haart L.G.J., Mertens J. (2015). Characterization of Ni-cermet degradation phenomena I. Long term resistivity monitoring, image processing and X-ray fluorescence analysis. J. Power Sources.

[57] Laurencin J., Quey R., Delette G., Suhonen H., Cloetens P., Bleuet P. (2012). Characterisation of Solid Oxide Fuel Cell Ni-8YSZ substrate by synchrotron X-ray nano-tomography: From 3D reconstruction to microstructure quantification. J. Power Sources.

[58] Boigues-Muñoz C., Pumiglia D., McPhail S.J., Santori G., Montinaro D., Comodi G., Carlini M., Polonara F. (2015). More accurate macro-models of solid oxide fuel cells through electrochemical and microstructural parameter estimation e Part II: Parameter estimation. J. Power Sources.

[59] Brus G., Miyawaki K., Iwai H., Saito M., Yoshida H. (2014). Tortuosity of an SOFC anode estimated from saturation currents and a mass transport model in comparison with a real micro-structure. Solid State Ionics.

[60] Kanno D., Shikazono N., Takagi N., Matsuzaki K., Kasagi N. (2011). Evaluation of SOFC anode polarization simulation using three-dimensional microstructures reconstructed by FIB tomography. Electrochim. Acta.

[61] Kishimoto M., Miyawaki K., Iwai H., Saito M., Yoshida H. (2013). Effect of composition ratio of Ni-YSZ anode on distribution of effective three-phase boundary and power generation performance. Fuel Cells.

[62] Brus G., Isomoto T., Iwai H., Saito M., Yoshida H., Komatsu Y., Nowak R., Szmyd J.S. Operating characteristics of an anode-supported planar SOFC stack with post-operation three-dimensional reconstruction of the electrodes microstructure. Proceedings of the 11th European SOFC and SOE Forum.

[63] Berg C.K. (2012). Re-examining Archie’s law: Conductance description by tortuosity and constriction. Phys. Rev. E.

[64] Berg C.K. (2014). Permeability description by Characteristic Length, Tortuosity, Constriction and Porosity. Transp. Porous Media.

[65] Hua B., Zhang W., Li M., Wang X., Chi B., Pu J., Li J. (2014). Improved microstructure and performance of Ni-based anode for intermediate temperature solid oxide fuel cells. J. Power Sources.

[66] Buyukaksoy A., Petrovsky V., Dogan F. (2012). Optimization of redox stable Ni-YSZ anodes for SOFCs by two-step infiltration. J. Electrochem. Soc..

[67] Fukui T., Ohara S., Mukai K. (1998). Long-term stability of Ni-YSZ anode with a new microstructure prepared from composite powder. Electrochem. Solid-State Lett..

[68] Pihlatie M., Kaiser A., Larsen P.H., Mogensen M. (2009). Dimensional behavior of Ni-YSZ composites during redox cycling. J. Electrochem. Soc..

[69] Hocker T., Iwanchitz B., Holzer L. Assessing the effect of electrode microstructure on repeat unit performance and cell degradation. Proceedings of the 7th Symposium on Fuel Cell Modeling and Experimental Validation (ModVal 7).

