# Supplementary material for: 3D Microstructure Effects in Ni-YSZ Anodes: Prediction of Effective Transport Properties and Optimization of Redox Stability
Source: Materials (Basel). 2015 Aug 26;8(9):5554–85. doi: 10.3390/ma8095265 (PMC5512617; doi:10.3390/ma8095265)
Supplement: Supplementary file 1 [file materials-08-05265-s001.pdf]

Supplementary Materials

Table S1. Particle size distributions of the NiO (*J.T. Baker*) and YSZ (*Mel Chemicals*) raw powders measured with a Horiba LA-920 laser diffractometer.

Raw Powder	d_{10} (µm)	d_{50} (µm)	d_{90} (µm)	d_{99} (µm)
NiO	0.34	0.62	2.02	3.18
8YSZ-fine	0.29	0.47	0.90	1.88
8YSZ-medium	1.09	3.33	6.42	9.88
8YSZ-coarse	6.01	10.19	15.82	22.50

Table S2. Summary of image data for 3D quantitative microstructure analyses of Ni-YSZ anodes before and after redox cycling.

State of Degradation	Anode Microstructure	Pixel Size (nm)		Slice Thickness (nm)	Pixel Matrix		Nr of slices	Total nr of Voxels	Size of image window (µm)			Total (µm^3)
		x	y	z	x	y	z		x	y	z	
Before redox	Fine	19.53	19.53	20.00	995	1'304	733	951'052'840	19.43	25.47	14.66	7'255.03
	Medium	24.41	24.41	25.00	960	1'110	610	650'016'000	23.43	27.10	15.25	9'682.77
	Coarse	29.14	29.14	30.00	744	1'417	456	480'737'088	21.68	41.29	13.68	12'246.39
After redox	Fine	19.53	19.53	20.47	1'171	1'343	461	724'993'033	22.87	26.23	9.44	5'660.52
	Medium	17.90	17.90	25.00	1'318	1'520	459	919'542'240	23.60	27.21	11.48	7'368.73
	Coarse	17.90	17.90	25.00	1'368	1'630	500	1'114'920'000	24.49	29.18	12.50	8'934.38

Table S3. Summary of image data for 3D analyses of simulated M-factors (M_{sim}) on GeoDict.

State of Degradation	Anode Microstructure	Pixel Matrix		Nr of Slices
		x	y	z
Before redox	Fine	500	500	500
	Medium	500	500	500
	Coarse	500	500	456
After redox	Fine	500	500	461
	Medium	1000	1000	459
	Coarse	1000	1000	500

Table S4. Summary of transport-relevant microstructure parameters and the predicted M-factors (M_{pred}) obtained using Equation (4).

	Before Redox Cycling			After Redox Cycling		
**Ni**	**Fine**	**Medium**	**Coarse**	**Fine**	**Medium**	**Coarse**
Φ	0.322	0.250	0.229	0.222	0.233	0.244
P	0.985	0.965	0.959	0.809	0.884	0.886
$\Phi_{eff} = \Phi \times P$	0.317	0.241	0.220	0.179	0.206	0.216
B	0.275	0.260	0.220	0.188	0.345	0.372
τ	1.219	1.341	1.605	1.375	1.358	1.673
M_{pred}	0.071	0.033	0.011	0.019	0.029	0.011
**YSZ**	Fine	Medium	Coarse	Fine	Medium	Coarse
Φ	0.421	0.388	0.384	0.312	0.376	0.324
P	0.999	0.986	0.923	0.961	0.869	0.184
$\Phi_{eff} = \Phi \times P$	0.421	0.383	0.354	0.300	0.327	0.060
B	0.367	0.095	0.007	0.088	0.042	0.0001
τ	1.108	1.176	1.889	1.430	1.353	1.100
M_{pred}	0.173	0.071	0.002	0.020	0.022	0.001
**Pore**	Fine	Medium	Coarse	Fine	Medium	Coarse
Φ	0.254	0.362	0.387	0.466	0.390	0.432
P	0.988	0.998	0.999	0.999	0.998	0.992
$\Phi_{eff} = \Phi \times P$	0.251	0.361	0.386	0.466	0.389	0.428
B	0.271	0.550	0.563	0.547	0.594	0.487
τ	1.324	1.110	1.103	1.073	1.081	1.082
M_{pred}	0.037	0.170	0.190	0.260	0.216	0.220

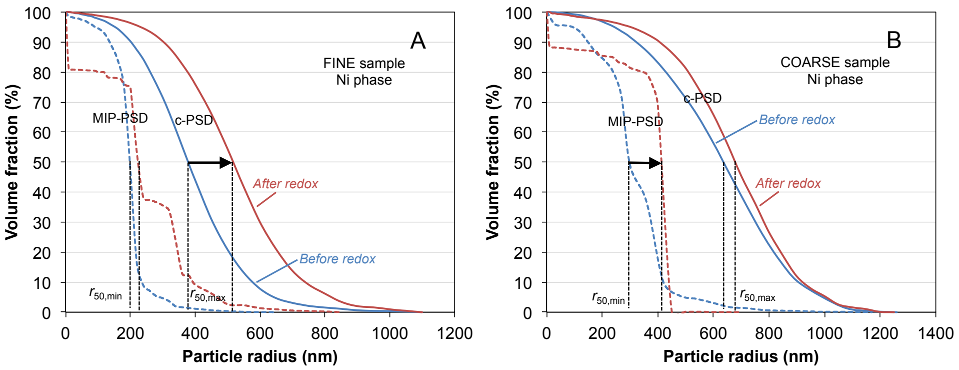

Figure S1. Representative c-PSD (solid lines) and MIP-PSD (dashed lines) before and after redox cycling. The c-PSD curves reflect the size of the bulges and the MIP-PSD curves reflect the size of the bottlenecks. (**A**) The shift in the c-PSD curve of Ni in fine sample after redox cycling illustrates the increase in the average size of the bulges while the MIP-PSD shows that the bottlenecks oft he Ni hardly change. (**B**) The YSZ bulges expand and the bottlenecks shrink in coarse sample as illustrated in the corresponding shifts in c-PSD and MIP-PSD curves.

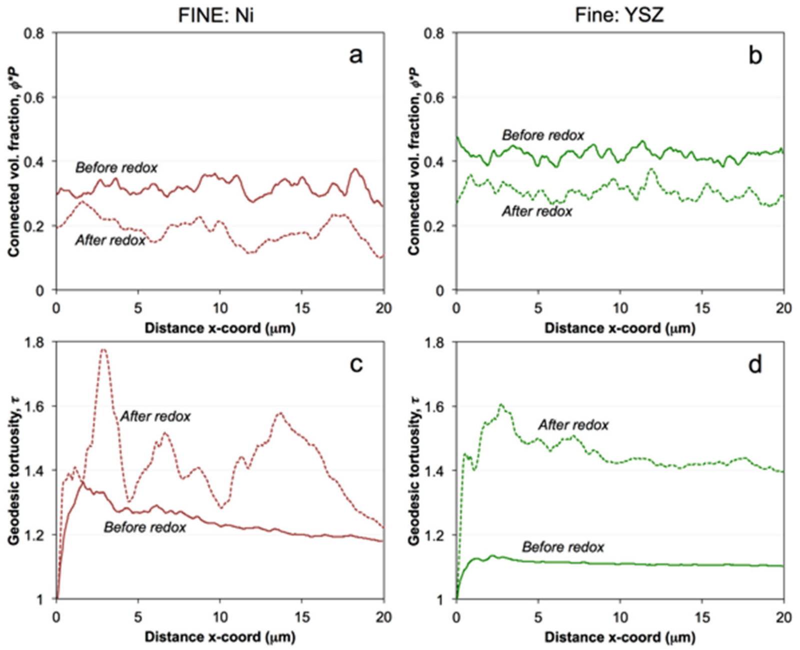

Figure S2. (**a**,**b**) Profiles of effective volume fraction ($\Phi \times P = \Phi_{eff}$) and (**c**,**d**) geodesic tortuosity (τ) in the fine anode as a function of the film thickness (x-direction). Note: Connectivity check is performed with the inlet plane on the left side (Distance 0). Connected volume fractions of Ni and YSZ in the fine sample vary in a narrow range. The effective (connected) volume fractions are slightly lower in samples after redox cycling, due to loss of percolation and swelling. Geodesic tortuosity lies between 1.1 and 1.35 before redox cycling, while higher (1.35–1.8) and more fluctuating tortuosities are observed after redox cycling.
